# Temporal transcriptome analysis reveals the two-phase action of florigens in rice flowering

**DOI:** 10.1007/s00122-025-04869-0

**Published:** 2025-04-12

**Authors:** Renwei Sun, Yifeng Ding, Manaki Mimura, Noriko Nishide, Takeshi Izawa

**Affiliations:** Laboratory of Plant Breeding and Genetics, Department of Agricultural and Environmental Biology, The University of Tokyo, Yayoi, Bunkyo-Ku, Tokyo 113-8657 Japan

## Abstract

Two florigen genes, *Hd3a* and *RFT1,* are essential for the floral transition in rice. To elucidate the early steps of the transcriptional dynamics during rice floral induction, we compared a set of temporal transcriptome data of SAM (shoot apical meristem)—region samples between the wild-type and a non-flowering line of the *hd3a rft1* double mutant during a short-day (SD) treatment after growing under long-day conditions for 42 days, and identified 6,978 DEGs (differentially expressed genes). As expected, *FUL-like* MADS-box genes were induced just after 4 days SD treatment; meanwhile, *SEP*-type and *AGL*-type MADS-box genes were induced after 9 days of SD treatment. We here newly revealed that majority of rhythmic genes including major circadian clock genes were not affected by the florigen genes, implying normal circadian clock phasing at the SAM regions regardless of floral transitions. We found that around two thousands of genes were repressed by *Hd3a* and *RFT1* genes at the SAM regions before the SD treatments and become derepressed and similar to WT expression levels in the double mutants according to the SD treatments. These clearly imply two distinct actions of florigen genes: one for repression of some developmental key genes during vegetative growth possibly by very low level of florigen expression and the other for floral induction by relatively high florigen expressions upon short-day inductions. This repression by low levels of florigens may serve as a maintenance system for vegetative growth before floral induction, which implies a novel role for florigen genes in rice.

**Supplementary Information:**

The online version contains supplementary material available at 10.1007/s00122-025-04869-0.

## Introduction

Flowering time in rice (*Oryza sativa*), a staple food for humans, is a critical agricultural trait that significantly influences both the quality and quantity of yield by enabling optimal harvest timing adapted to local conditions. The timing of floral transition in rice is influenced by the genetic background of cultivars and the cultivation environments to which they are exposed. It is well known that florigen genes encoding a mobile flowering signal protein are transcribed under the inductive environments, translated in the leaves, and the translated proteins are moved to the apex regions of plants (Corbesier et al. 2007; Tamaki et al. 2007; Turck et al. 2008).

Florigen is approximately 20-kDa globular protein belonging to the phosphatidylethanolamine-binding protein (PEBP) family, with a conserved and systemic function (Kardailsky et al. 1999; Turck et al. 2008). In rice, *HEADING DATE 3a (Hd3a)* and *RICE FLOWERING LOCUS T1 (RFT1)* are recognized as florigens, as RNAi knockdown and CRISPR/Cas9 mutants of these genes result in non-flowering phenotypes (Komiya et al. 2008; Mineri et al. 2023). These two genes are located linearly on chromosome 6, separated by only 11.5 kb, and share 91% sequence similarity (Kojima et al. 2002; Komiya et al. 2008, 2009). Despite their proximity and high sequence similarity, *Hd3a* and *RFT1* play distinct roles in regulating rice flowering. *Hd3a* is specifically induced under short-day (SD) conditions by detecting subtle changes in day length of 30 min or more (Itoh et al. 2010). In contrast, *RFT1* is more highly expressed under long-day (LD) conditions and plays a more prominent role in determining flowering time in temperate regions (Itoh et al. 2010; Izawa et al. 2002; Ogiso-Tanaka et al. 2013).

Molecular genetic analysis over the past decade has revealed both an evolutionarily conserved pathway which is shared among plant species and a monocot-specific pathway to control transcriptional regulation of both *Hd3a* and *RFT1* in rice (Izawa 2021). The conserved pathway consists of *OsGI–Hd1–Hd3a/RFT1* pathway (analogous to the *GI–CO–FT* pathway in *Arabidopsis*); meanwhile, a monocot-specific pathway consists of *Ghd7- Ehd1 – Hd3a/RFT1* pathway, where it is known that Hd1 and Ghd7 proteins make a transcriptionally repressive protein complex to control *Ehd1*. It is of note that blue light induction of *Ehd1* in the morning requires *OsGI* function (Itoh et al. 2010; Izawa et al. 2011). Recently, the *Ghd7* pathway has been shown to be the target of ambient temperature signaling (Nagalla et al. 2021).

With short-day inductions, translated Hd3a and RFT1 proteins move from leaves to the SAM (shoot apical meristem) regions to start floral induction out there. Once florigen proteins are transported to the SAM regions, both two florigen molecules bind with two molecules of 14-3-3 proteins in the cytoplasm, forming a tetramer. This tetramer is subsequently translocated to the nucleus, where it binds with two phosphorylated *OsFD1* bZIP transcription factors to create a heterohexameric complex, known as the florigen activation complex (FAC) (Peng et al. 2021; Taoka et al. 2011). The florigen-GF14-OsFD1 complex, leveraging the DNA-binding functionality of the bZIP transcription factor, binds directly to the C-box motif (GACGTC) in the promoter region of the target gene *OsMADS15*, thereby initiating inflorescence development (Taoka et al. 2011). Further genome-wide studies using DAP-Seq analysis have identified additional potential targets for FAC (Cerise et al. 2021). Then, multiple studies have shown that alternative FACs may form with other bZIP proteins, potentially incorporating one or both florigen molecules. Furthermore, some bZIP-florigen interactions occur without the need for GF14 as a bridging component (Cerise et al. 2021; Kaur et al. 2021), as observed with flowering-repressing bZIP transcription factors such as Hd3a BINDING FACTOR 1 & 2 (HBF1&2) (Brambilla et al. 2017). Even in leaves, *Hd3a* and *RFT1* can form a standard FAC with *OsFD1* and *Gf14c* to activate *Ehd1* expression, while they can also inhibit *Ehd1* expression by interacting with HBF proteins (either HBF1 or HBF2). *Hd3a* can interact directly with HBFs, whereas *RFT1* requires GF14c to interact with HBFs indirectly. After *Hd3a* and *RFT1* are transported to the meristem, they can promote the transcription of target genes *OsMADS14/15* by forming the standard FAC, whereas HBF1 can repress the transcription of the same targets through the formation of an inhibitory FAC (Brambilla et al. 2017). Moreover, triple mutants of *OsFD1 OsFD3*, and *OsFD4* only exhibited partially delay flowering, indicating potential gaps in the current FAC model (Cerise et al. 2021; Izawa 2021).

In addition to the two primary florigen proteins, around ten *FT-like* genes exist in rice (Izawa et al. 2002; Zheng et al. 2023). In addition, four other PEBP family members, known as *TERMINAL FLOWER 1* (*TFL1*) *-like* genes, *RCN1-4* genes, also play a role in flowering regulation in rice (Zheng et al. 2023). These genes are commonly referred to as anti-florigen proteins (Conti and Bradley 2007; Nakagawa et al. 2002). At the molecular level, they often antagonize the function of *Hd3a* and *RFT1*, competing to bind with GF14 and forming a florigen repression complex (FRC) that delays the transition to reproductive growth (Ahn et al. 2006; Kaneko-Suzuki et al. 2018). Similar FRCs have been identified in other *FT-like* proteins, such as *OsFTL12* (Zheng et al. 2023). Moreover, the transport of florigen proteins *Hd3a* and *RFT1* to the SAM activates *OsFT-L1*, another *FT-like* gene at SAM in rice (Giaume et al. 2023).

During the reproductive transition, the SAM region undergoes an active identity shift and SAM itself gradually elongates upward, accompanied by stem elongation, which ultimately influences panicle architecture in rice. In this process, florigens mediate gibberellin sensitivity in the stem by downregulating *PREMATURE INTERNODE ELONGATION 1* (*PINE1*), leading to stem (or internode) elongation (Gomez-Ariza et al. 2019). *BRT1*, another downstream target gene of both florigens, has a significant impact on leaf angle and panicle development (Mineri et al. 2023). Furthermore, the regulatory pathway involving the *miR156/529-SPLs-NL1-PLA* module plays a crucial role in floral transition, bract suppression, and spikelet branching (Wang et al. 2021). However, its relationship with the FAC remains unclear.

Previously, RNA-seq analysis explored the downstream gene network regulated by florigen in the SAM of five-year-old florigen double mutants, revealing that FT-like proteins induce transposon silencing in the shoot apex during floral induction (Tamaki et al. 2015). In addition, a similar study has also investigated gene expression changes in the SAM mediated by individual *Hd3a* or *RFT1 *(Mineri et al. 2023), yet these analyses lack a comprehensive identification of downstream regulators upon inflorescence meristem formation.

In this study, we aim to identify florigen-regulated target genes at the SAM regions during the early stages in floral transition (including the inflorescence transition phase). Thus, we conducted a set of time-course RNA-seq experiments, systematically sampling the SAM regions (containing SAM and a few leaf primordia) at various developmental stages during SD treatments to compare gene expression between non-flowering plants of a florigen double mutant line and the wild-type plants. This temporal analysis captures gene expression dynamics throughout floral transition, providing a comprehensive view of florigen’s regulatory role. Our results reveal the two-phase action of florigens during rice floral induction.

## Materials and methods

### Plant materials and growth conditions

For this study, we used a rice *temperate japonica* cultivar named Koshihikari as the wild-type control and a florigen double mutant line (MU) with Koshihikari background. The original mutant line was obtained from lines transformed with T-DNA possessing both CRISPR-Cas9 and sgRNA expression units. Here, the sgRNA sequences were CCTGGGCTGTTGGGTCACCA (For *RFT1*) and GGCTGGTGGGTGACCATGGA (For *Hd3a*). Rice seeds were surface sterilized by immersion in a 20% sodium hypochlorite solution for 30 min, thoroughly rinsed with sterile water, and then placed in a plant growth chamber under dark conditions for 2 days to promote germination. Given that the florigen double mutant plants are incapable of flowering, the F_2_ seeds used in this experiment were obtained from heterozygous F_1_ plants. Genotyping was conducted to distinguish the segregating genotypes among F_2_ seedlings, using molecular markers specific to the 45 bp and 18 bp indels in *Hd3a* and *RFT1*, respectively. This genotyping process made us confirm that no related extra flowering time and other morphological mutations are not involved in these experiments since non-flowering phenotypes unlinked to florigen genes were not observed yet. For plant growth, both long-day (LD) and short-day (SD) photoperiod conditions were subjected as LD: light 9:00 AM—11:30 PM, resulting in a 14.5-h light/dark cycle, with day 28 °C and night 25 °C; SD: light 9:00 AM–7:00 PM, resulting in a 10-h light/dark cycle, with day 28 °C and night 25 °C.

Sampling was performed to collect the shoot apical meristem (SAM) regions including a few leaf primordia from the main stem of three individual plants. To access the SAM regions, the outer leaf tissues were carefully removed as much as possible, and the SAM was excised and immediately placed in a tube for preservation. The excised SAM samples were flash-frozen in liquid nitrogen to maintain RNA integrity. Three SAM samples from the main stems of three plants were collected into a sample tube (the first tube). Another three SAM samples from the main stems of three different plants were collected into another tube (the second tube) in the same manner as the first tube to serve as a biological replicate.

### Total RNA isolation and RNA-seq experiments

Total RNA was extracted from the collected SAMs using the TRIzol reagent (Invitrogen, Waltham, MA, USA), following the manufacturer’s protocol. The extracted RNA was enriched for mRNA to construct transcriptome libraries. Library preparation was performed using the VAHTS® Universal V8 RNA-seq Library Prep Kit for Illumina (NR605; Vazyme, Nanjing, China), in accordance with the supplier’s instructions.

Library quality assessment and sequencing were conducted by Macrogen Japan, resulting in the acquisition of raw sequencing data. Sequencing was performed on a HiSeqX Ten platform in a 150-bp paired-end configuration. Quality control and read trimming were carried out using FASTP (version 0.23.4; Chen et al. 2018). The high-quality reads were aligned to the *Oryza sativa* Japonica Group cultivar Nipponbare IRGSP-1.0 reference genome (Rice Annotation Project Database, RAP-DB, http://rapdb.dna.affrc.go.jp) using STAR (version 2.6.1b) (Dobin et al. 2013). Following alignment, reads were assigned to transcript IDs using the FEATURECOUNTS function in the SUBREAD package (version 1.6) (Liao et al. 2014). Detailed statistics for raw and trimmed reads for each sample are provided in the mapping summary (Table S1). Subsequent data analysis was performed in R (version 4.6). Gene counts were normalized for sequencing depth and transformed into TPM values using the edgeR package (version 4.4.0) (Robinson et al. 2010). Principal component analysis (PCA) was conducted using prcomp in R and visualized with ggplot2 (version 3.3.3; https://ggplot2.tidyverse.org).

### Differential expression (DE) analysis

After performing quality control on the sequencing data using Fastp, the average reads count across all SAM samples was 21,564,812 (> 20 M). On average, 91.25% of the reads were uniquely mapped to the reference rice genome (RAP-DB). Non-specific reads were discarded to eliminate potential ambiguities. These metrics indicate that the preprocessing parameters were suitable for downstream analysis (Table S1). DE analysis was conducted using the edgeR package with two biological replicates per condition. Transcripts with an absolute log2 fold change greater than 1.0 and an adjusted p value (FDR) < 0.05 were considered differentially expressed. For each time point, DEGs were identified as described above and then combined using a union approach to integrate all DEGs across stages.

### Data processing

Heatmaps were generated using the R package ClusterGVis (version 0.0.4) (https://github.com/junjunlab/ClusterGVis), with clustering based on the Mfuzz algorithm. Additionally, expression trend line plots were generated to illustrate gene expression trajectories, connecting the mean TPM values across conditions. The identification of rhythmic gene expression in the SAM was based on TPM values over multiple complete cycles, using MetaCycle’s integration method with an FDR threshold of less than 0.05 (Wu et al. 2016). Clustering was performed using the Mfuzz method.

### In situ hybridization

F_2_ plants were grown under identical conditions to the RNA-seq material, followed by genotyping to distinguish between the wild-type and the double mutant plants. Sampling times matched those for RNA-seq, with one shoot apex collected from each seedling. The shoot apices were dissected, fixed in 4% paraformaldehyde in 0.1 M sodium phosphate buffer for overnight at 4 °C, and then dehydrated in a graded ethanol series. Ethanol was replaced with Histo-Clear (National Diagnostics, Atlanta, GA), and samples were embedded in Paraplast Plus (Leica, Wetzlar, Germany). Paraffin Sects. (8 μm thick) were mounted on slides coated with 3-aminopropyl triethoxysilane (Matsunami Glass, Osaka, Japan).

To prepare probes, first-strand cDNAs were synthesized from total RNA using ReverTra Ace qPCR RT Master Mix (TOYOBO, Osaka, Japan). Partial coding sequences of each gene were amplified from cDNA by PCR using the primers including T7 promoter sequence as listed in Table S1. Digoxigenin (DIG)-labeled antisense RNA probes were generated by in vitro transcription using T7 RNA polymerase (Takara, Shiga, Japan) and DIG-RNA labeling mix (Roche). In situ hybridization and immunological detection of hybridization signals were conducted as described (Miya et al. 2025).

## Results

### Rice florigen double mutants did not respond to the short-day (SD) treatments

**Fig. 1:**
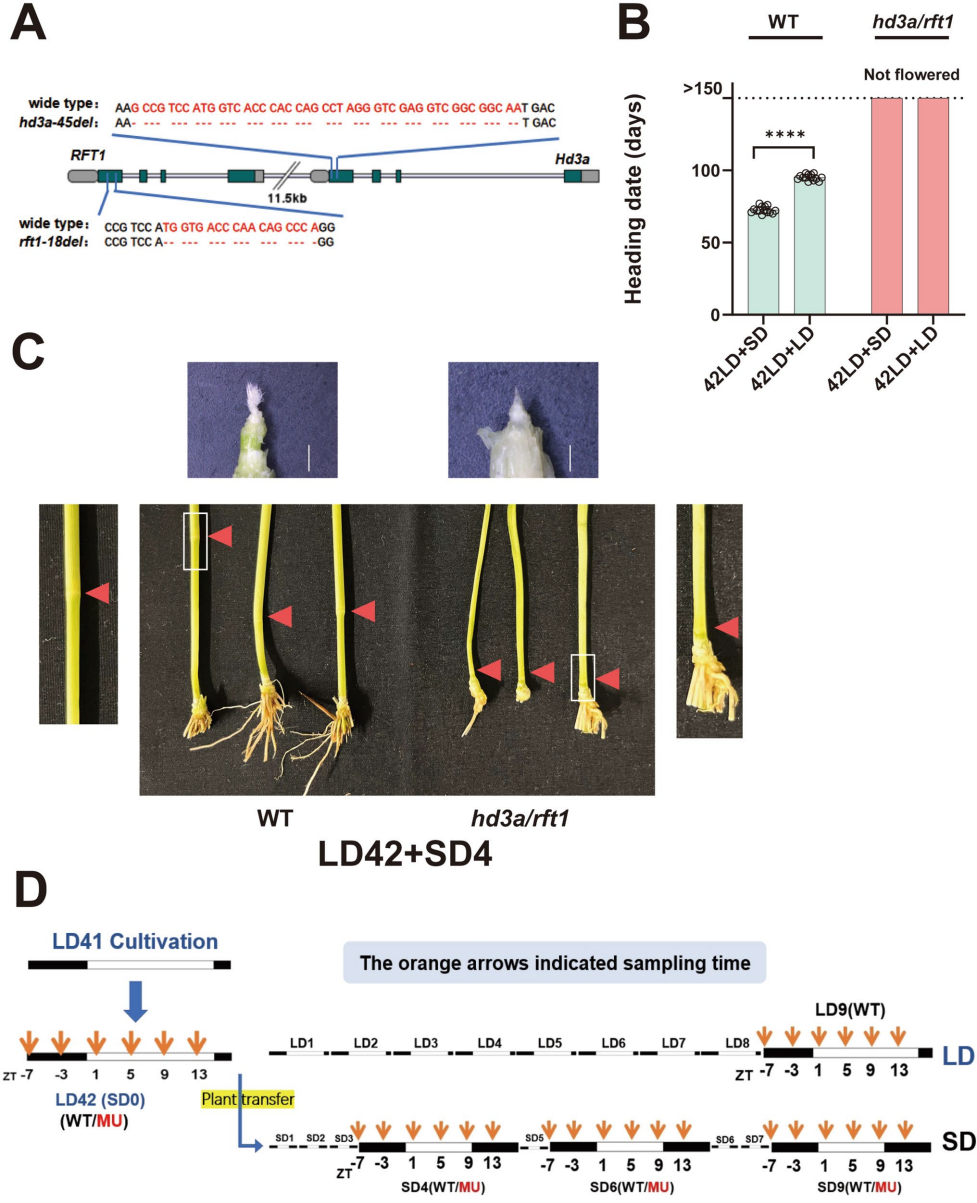
The double mutants of rice florigen genes exhibited non-flowering and did not respond to photoperiods. **A** The 45 bp and 18 bp deletions at the first exon of *Hd3a* and *RFT1,* respectively, in the original double mutant line. **B** Flowering time of the wild-type and the florigen double mutants. The plants were grown for 42 days under the long-day conditions and then subjected to SD treatments. *n* = 18. On flowering-time of WT: *p* < 0.0001 by Student’s *t* test (two-tailed). **C** Time schedules for SAM sampling. The wild-type and mutant samples were sampled as indicated. The sampling started at 42 days after sowing. Sampling timings are illustrated by orange arrows. White and dark bars denote the light and dark periods, respectively. Sampling was conducted six times over a day, with intervals of four hours. **D** Phenotypic changes of wild-type and the double mutant plants at SD4. Stem elongation started in the wild-type plants, but no elongation in the double mutant plants. Bars represent 2 mm. The location of the SAM is marked with a white box. Red arrows indicate the node positions. Photographs of SAM regions after removal of surrounding leaves are also shown

Utilizing a *temperate japonica* rice cultivar ‘Koshihikari’ as the genetic background, we employed CRISPR-Cas9 gene-editing technology and resulted in the introduction of some deletions in both *Hd3a* and *RFT1*, excising 45 bp/6 bp and 18 bp from the first exon of each gene, respectively (Fig. 1A), and confirmed non-flowering phenotypes around one year in growth chambers only for the double mutant plants among self-pollinating progenies of the regenerated T0 plant. Through the crossing with the wild-type plants, we obtained a F_1_ heterozygous plant where one of chromosomes harbored both tandemly aligned mutations of *Hd3a* and *RFT1*, while the other chromosome remained intact. Subsequent self-pollination of these F_1_ plants allowed to select seedlings of the double homozygous mutant for both genes.

To assess the flowering response, we initiated a short-day (SD; 10 h light and 14 h dark) treatment after 42 days of long-day (LD; 14.5 h light and 9.5 h dark) cultivation. Wild-type rice plants flowered around 70 days with the SD treatments compared to around 90 days under the continuous LD conditions (Fig. 1B). Considering that it would take around 30 days from floral induction to heading, the floral transition was likely to start just after SD induction; meanwhile, the floral transition at LD42 (42 days grown under long-day cultivation) (or SD0 (0 day after the short-day induction)) did not start in these experiments. In wild-type plants, rapid upward growth of the shoot apical meristem (SAM) was evident after the SD induction, marking the inflorescence transition and stem elongation also started at SD4 (Fig. 1C). In contrast, the double mutant plants did not flower even after 150 days, showing non-flowering phenotypes even after the long SD treatment. The SAM in the double mutant plants remained at the base regions during the SD treatment, indicating that florigen is involved in stem elongation in rice as previously reported (Gomez-Ariza et al. 2019) (Fig. 1B, Fig. 1C).

To investigate the different transcriptomic dynamics among the specific stages of floral induction, we designed time-course RNA-seq experiments. As shown in Fig. 1D, we collected SAM region samples of both the wild-type and the double mutant plants across four days (SD0, SD4, SD6, and SD9), with six time points per day (ZT-7, ZT-3, ZT1, ZT5, ZT9, and ZT13), to capture a comprehensive transcriptional landscape of floral induction dynamics. At the same time, time-series sampling was also performed only for the wild-type plants under long-day conditions at LD9 (42 + 9 days grown under the LD conditions) to confirm the floral transition state under the continuous LD conditions (Fig. 1D).

### Mapping of time-course DEGs with previous knowledge

**Fig. 2:**
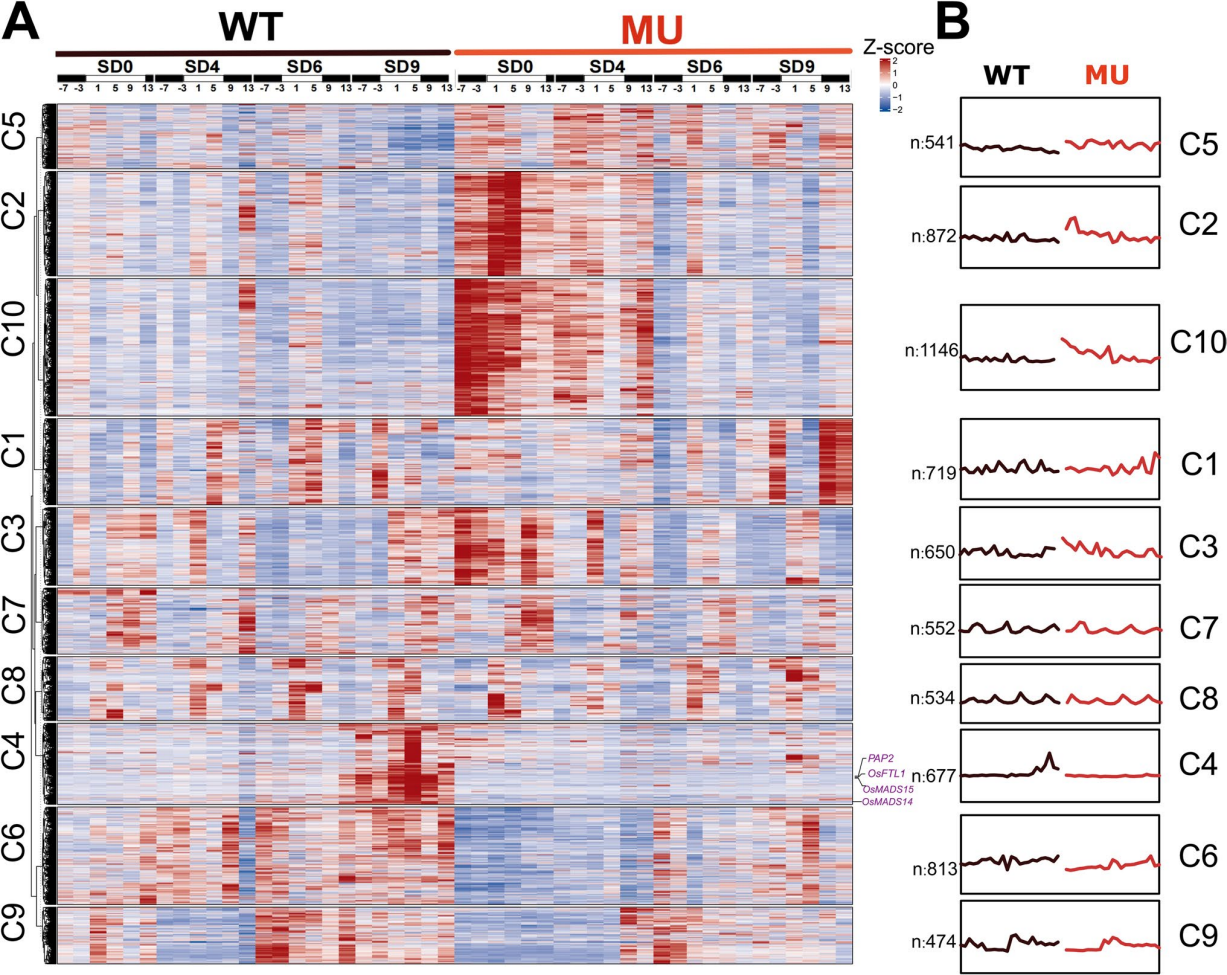
Panoramic display of all identified differential expression genes. **A** The temporal expression profiles of all 6,978 differentially expressed genes were plotted across 24 sampling time points with six time points per day (ZT-7, ZT-3, ZT1, ZT5, ZT9, ZT13). The heatmap was generated based on the TPM of each gene expression, standardized using z-scores. Clustering was performed using Mfuzz method, and representative genes in cluster4 were labeled. **B** The numbers represent the gene number count in each cluster, and in the line charts showing expression trends, the lines connect the TPM-standardized mean values. Black line (WT), wide type; red line (MU), double mutant

A comprehensive, temporal map of differentially expressed genes (DEGs) in the SAM has been generated to illuminate gene expression changes associated with floral induction in rice (Fig. 2A). By applying stringent selection criteria for differential expression (∣log_2_(fold-change)∣ > 1, FDR < 0.05), we compiled the DEG sets based on the wild-type (WT) versus the double mutant line (MU) comparisons across all 24 (6 time points × 4 days) samples, yielding a total of 6,978 unique DEGs (Fig. 2A). The expression trajectories of these 6,978 genes in both WT and MU were then visualized to highlight differential patterns across the timeline (Fig. 2B). These DEGs at the SAM regions span the critical phases of floral meristem differentiation in rice and were categorized into 10 distinct expression clusters based on expression levels and patterns. Cluster10 (C10), for instance, is characterized by a prominent upregulation of gene expression in the MU, housing the largest number of genes showing enhanced expression in the double mutant line. Genes annotated with floral regulators such as *FUL-like MADS box* (*OsMADS14/15*) and *PAP2 *(*OsMADS34*) genes predominantly populated in C4, indicating a specific expression pattern associated with floral differentiation markers. The analysis also encompassed samples collected at four distinct days (SD0, SD4, SD6, and SD9), representing different developmental stages of floral transition. Each day sample revealed a unique DEG profile: 4213 DEGs in SD0 (pre-induction) exhibiting the highest number. In addition, SD4, SD6, and SD9 displayed 3,039, 1,207, and 3,646 DEGs, respectively (Fig. S1).

**Fig. 3:**
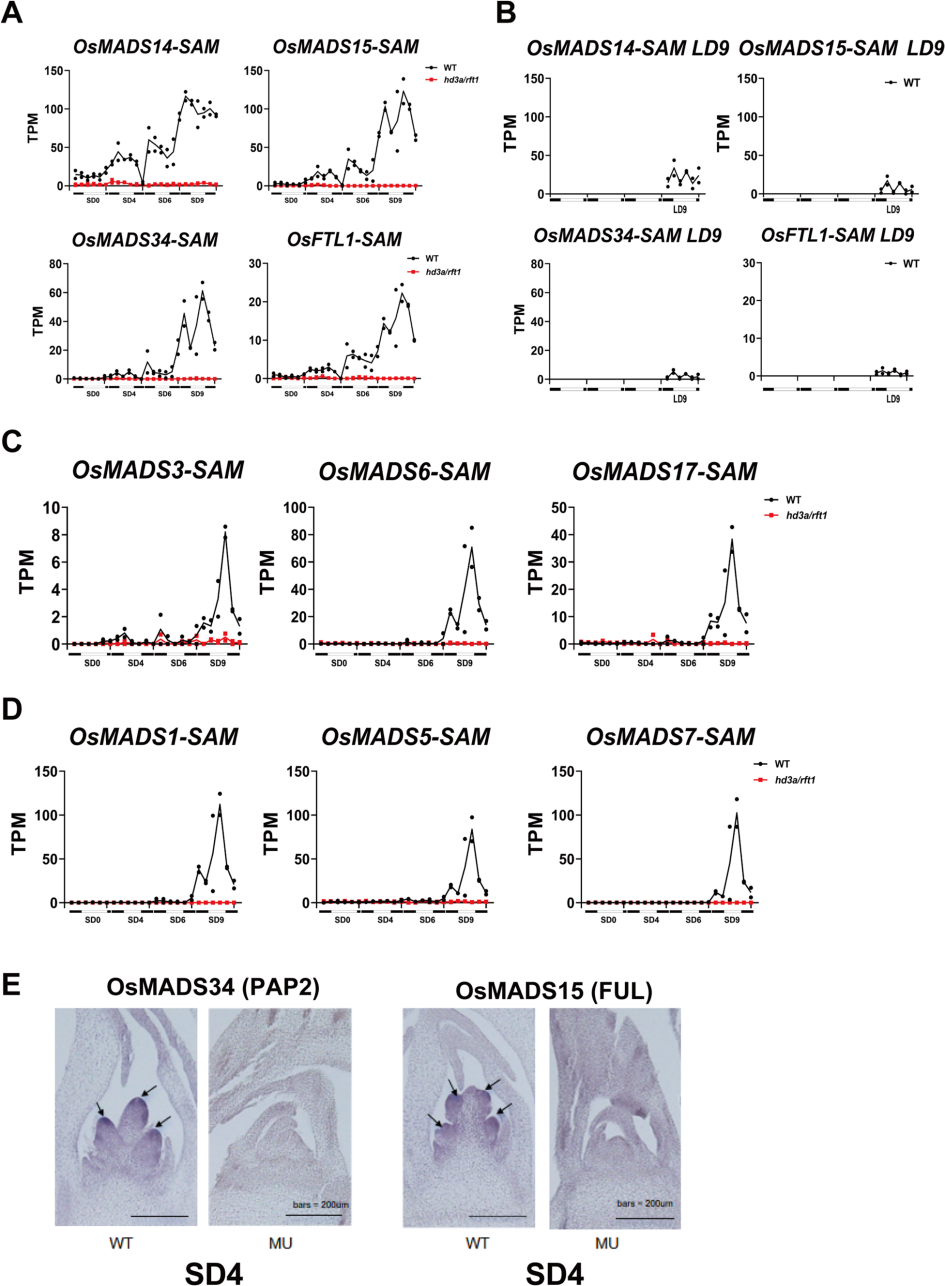
Expression analysis of representative floral differentiation genes in SAM. **A** The expression patterns of four representative genes induced by SD treatments are shown. The lines connect the mean TPM values of two replicates. Black dots and lines represent the wild type, and red dots and lines represent the double mutant; this color scheme applies to all subsequent graphs. Photoperiod conditions are indicated on the x-axis. **B** Expression analysis of four representative genes in SAM under LD9 conditions. **C** Expression analysis of *AGL* genes in SAM. **D** Expression analysis of *SEP-like* genes in SAM. **E** Gene expression of *OsMADS15(FUL)* and *OsMADS34(PAP2)* in SAMs produced by in situ hybridization. Bar = 200 μm. Two times of the experiment were repeated with similar results. Arrows indicate branch meristems

First, we confirmed how our temporal transcriptome data are consistent with the previous knowledge in this field. It is known that short-day induction initiates phase changes in SAM from vegetative meristem (VM) to inflorescence meristem (IM), which has been believed to be primarily driven by the activation of *FUL-like* MADS-box transcription factors. Genes such as *OsMADS14* and *OsMADS15* (both orthologs of the *FUL* gene in *Arabidopsis thaliana*) exhibit substantial induction, marking them as pivotal players in the onset of IM differentiation. In this study, we observed a progressive “uphill” expression trend for these genes according to the SD treatments in the wild-type plants, while in the double mutant line, they remained consistently unexpressed (Fig. 3A). As expected, we observed the starts of expressions for *OsMADS15/ PAP2 (OsMADS34)/FTL1* at SD4, but not at SD0; meanwhile, no expression was observed in MU at SD0, SD4, SD6, and SD9 (Fig. 3A). For *OsMADS14*, we observed some expressions in WT but not in MU. The expression of these genes was also analyzed with LD9 samples, showing critically reduced expression relative to SD4 samples (Fig. 3B). This observation aligns with the phenotype data in Fig. 1B, where LD delays IM differentiation and flowering in the wild-type plants, confirming that the floral formation was not yet started at LD9. These results suggest that the developmental stages of samples at SD4 are related to the IM stages. This conclusion is supported and revised a bit by in situ hybridization results at SD4 as the wild type had already initiated inflorescence differentiation at SAM regions, with high expression of *OsMADS15* and *PAP2*, while the mutants remained no expression (Fig. 3E). However, our in situ analysis also tells us that primary branch formation already started in samples at SD4, indicating that RNA samples at SD4 may strongly associate with primary branching stages rather than IM transition itself. In this study, we may miss the onset stage samples for IM transition.

The development and morphology of floral organs are a highly complex process involving numerous factors and their interactions. Specifically, during floral organogenesis, *AGAMOUS-LIKE(AGL)* family genes regulate stamen and carpel formation, as exemplified by *OsMADS3/6/17*, while *SEPALLATA (SEP)* family genes function as cofactors, contributing to the development of various floral organs (Becker and Theissen 2003; Chung et al. 1994). In our study, we observed the expression of *AGL* and *SEP* family members in the wild type primarily at SD9, aligning with the onset of floral organ formation (Fig. 3C, D). This mapping provides robust support for the biological validity of our sequencing data.

We also investigated the expression patterns of core regulatory genes involved in rice flowering. Notably, florigen itself is not expressed in the SAM (Fig. S2), as it has previously shown that leaves are the primary site of florigen expression. However, among the *FTL* and *TFL* family members, only *OsFTL1* and *OsFTL4* are expressed in the SAM region (Fig. 3A, Fig. S3), although *OsFTL4* showed high expression levels even in the double mutant line. Other *FTL* genes were not detected in the SAM; hence, two of them, *OsFTL6* and *OsFTL11,* are highlighted as representative genes (Fig. S3). Meanwhile, *TFL* subfamily genes, *RCN1* ~ *4*, and *OsMFT1* were expressed in the SAM of both WT and MU lines although *OsMFT2* was not in the SAM (Fig. S3). Furthermore, we also examined upstream regulators of florigen, such as *Hd1*, *Ehd1*, and *Ghd7*. The expression levels of these genes in the SAM were either very low or did not show significant expression, suggesting limited involvement in floral initiation at the SAM regions during floral induction (Fig. S4A–C). It is noteworthy that *OsCOL4*, a flowering-time gene, was rhythmically expressed in the SAM regions but not affected by florigen genes.

Next, we examined data on currently reported members of the FAC, including paralogous genes of *OsFD1* (*OsbZIP77*) and various GF14 family members (Fig. S5A, B). These components are known to play key roles in forming the FAC with florigen, thereby possibly related to the initiation of inflorescence differentiation. Our analysis revealed that the majority of these FAC components exhibited similar expression levels between the wild-type and the double mutant lines. Interestingly, *OsFD1* exhibited decreasing expression in WT, thus relatively higher expression in MU according to the SD treatments. Only a few other members showed differential expression at specific time points, such as *OsFD4*, which demonstrated a notable expression variance at the SD4 time point (Fig. S5A, B). Overall, these data suggest that the expression of FAC members remains largely unaffected by the presence of functional florigen genes, indicating that florigen deficiency does not significantly alter the FAC expression profiles. The *miR156/529-SPLs-NL1-PLA* pathway plays a pivotal role in the transition of the inflorescence (Wang et al. 2021). In this study, we also investigated the impact of florigen deficiency on genes within this pathway. Interestingly, we found that the expression levels of core *SPL* genes remained unaffected in the florigen double mutant (Fig.S6), suggesting that the regulation of flowering time via the *miR156/529-SPLs* pathway operates independently of the florigen-regulated pathway. In addition, we also examined genes involved in SAM formation and maintenance such as *OSH1*. These genes are critical for the establishment of final inflorescence architecture in addition to the development of SAM. Notably, we observed that *APO2* displayed higher expression levels in the wild-type plants than those in the double mutant lines, whereas *PINE1*, *BLH*, and *OSH15* were markedly upregulated in the double mutant lines (Fig. S7). Those regulation observed in the double mutant plants suggests that the absence of florigen may change the expression of these SAM-maintaining genes, likely altering the initiation of floral transitions and the morphological outcomes of the inflorescence in rice.

### Core circadian clock genes and their downstream genes remain unaffected in SAM by loss of florigen function

Over the past decades, circadian clock genes have been extensively studied, given their role as core regulators of the biological clock that modulates a wide range of physiological processes, including the timing of flowering (Gombos et al. 2023; Izawa et al. 2011). However, the presence and function of circadian rhythm genes within the shoot apical meristem have yet to be thoroughly explored, even though the SAM is the critical site where the flowering process is ultimately initiated. Thus, we next investigated the circadian gene network within the SAM regions in rice, utilizing MetaCycle to identify diurnally rhythmically expressed genes in the SAM region.

**Fig. 4:**
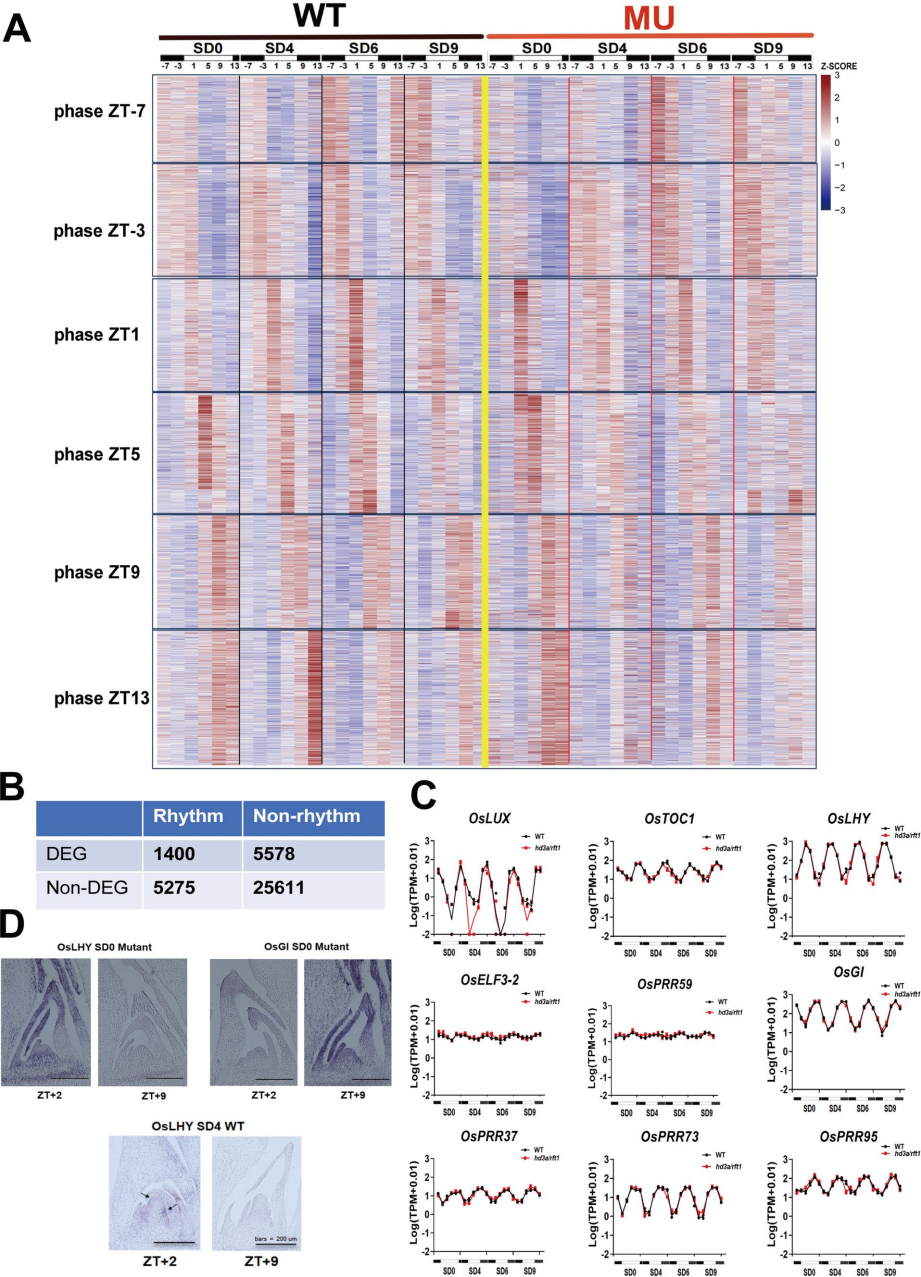
A comprehensive map of rhythmic genes identified in SAM. **A** Based on MetaCycle analysis, a total of 6,675 rhythmic genes were identified in SAM. The heatmap was generated based on the TPM of each gene expression, standardized using z-scores. **B** The table describes the number relationship between rhythmic genes and differentially expressed genes (DEGs). **C** Expression analysis of representative circadian clock genes in the wild type and the double mutant. **D** The expression of *OsLHY* and *OsGI* in SAM was validated through in situ hybridization experiments. Bar = 200 μm

Through this analysis, we identified a total of 6,675 diurnally rhythmic genes within the SAM, displaying a diverse array of rhythmic expression patterns. The expression patterns of the rhythmic & non-DEG (*n* = 5275) and rhythmic & DEG (*n* = 1400) gene groups were together visualized according to peak positions among the six time points (phases) within a day (Fig. 4A). Interestingly, only approximately one-fifth (1,400 out of 6,675) of these genes were DEGs by florigen functions (Fig. 4B), indicating that most diurnally rhythmic genes in the SAM regions remain unaffected by the absence of florigen. This finding supports the notion that most circadian clock genes operate upstream of florigen and are not directly affected by florigen genes. Their diurnal rhythm patterns varied continuously. For example, *OsLHY and OsGI* had a peak and trough at ZT1 in the morning, respectively (Fig. 4C). In addition, *OsGI*, *OsTOC1*, and *PRR* family members (*OsPRR59*, *OsPRR37*, *OsPRR73*, and *OsPRR95*) had peaks in the evening. Importantly, the expression levels of these core circadian clock genes showed no significant differences between wild-type and the double mutant lines (Fig. 4C). These rhythmic expression patterns were further validated through in situ hybridization (Fig. 4D), confirming the diurnal regulation of circadian clock genes within the SAM although it becomes an open question how weak lights at the SAM regions can entrain the circadian clocks properly although peak phases of some genes were likely affected by the florigen genes (Fig. 4A).

Therefore, we have also analyzed the expression patterns of several photoreceptor genes, including *PHY A, B* and *C, CRY1A, 1B*, and *NPH1A*, in the SAM region (Fig. S8). While they did not exhibit clear rhythmic expression, *PHYA* showed derepressed expression in the florigen-deficient mutant compared with the wild type. In addition, *NPH1A* did not express at the SAM region. In contrast, other photoreceptors remained largely unaffected by the loss of florigen function, indicating that phytochrome and cryptochrome photoreceptors, but not *NPH1A* photoreceptor, may work even at the SAM regions. Thus, weak lights at the SAM region may entrain the circadian clocks. These data may also highlight a potential specific relationship between florigen and *PhyA* genes (Takano et al. 2005), where the loss of florigen may increase the *phyA* signal sensitivity. But we need to examine this possibility in future.

To gain deeper insight into the rhythmicity of the rhythmic genes in the SAM regions, we conducted principal component analysis (PCA) on all identified rhythmic genes, as well as on the subset of 1,400 genes that are both rhythmic and differentially expressed (Fig. S9). These PCA maps demonstrate that samples collected at different time points displayed a circular distribution, reflecting the proper timekeeping in the rice SAM regions without clear entrainment signals in the SAM regions. For the subset of 1,400 rhythmic DEGs, including many genes related to the floral transitions, the PCA analysis revealed that samples from most time points (except for SD6) showed clear separations between the wild-type and the double mutant lines, while still preserving their rhythmic expression dynamics (Fig. S9). This study highlights the independence of circadian clock regulation even in the SAM regions and the existence of a robust, diurnal rhythmic gene expression network that persists regardless of the florigen functions.

### A Hidden transcriptional regulation by florigens in rice

**Fig. 5:**
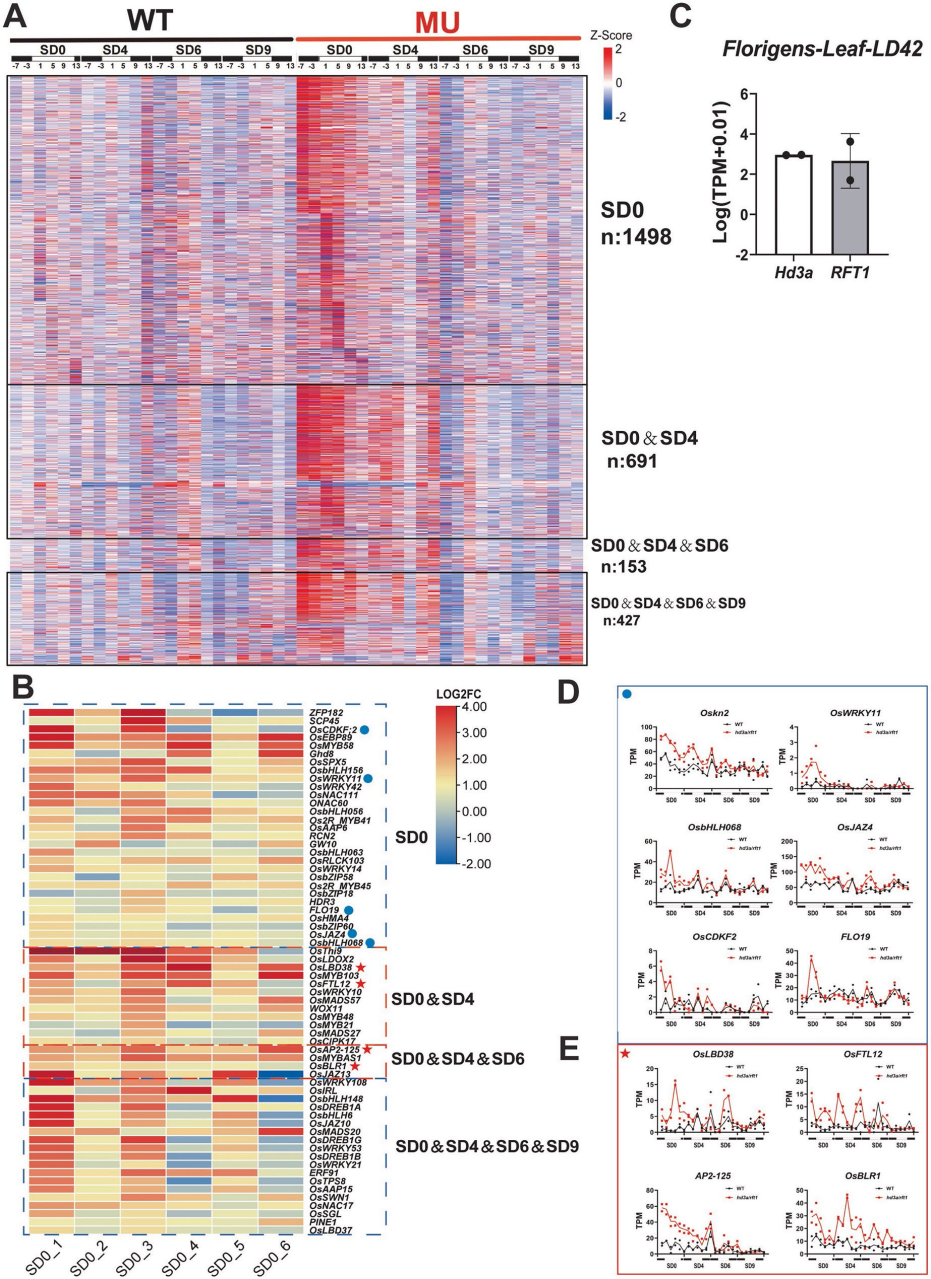
The high expression of regulators in the SAM at SD0. **A** Genes with expression changes at the SD0 were visualized in a heatmap, with expression levels presented based on normalization and arranged in the following order: ① 1,498 genes that were DEGs only at SD0; ② 691 genes that were DEGs at both SD0 and SD4; ③ 153 genes that were DEGs at SD0, SD4, and SD6; ④ 427 genes that were DEGs at SD0, SD4, SD6, and SD9. **B** Several genes with differential changes of expression were selected to create a heatmap. The heatmap displays the fold change (FC) values at each time point in SD0. **C** Even under LD conditions (SD0 or LD42), *Hd3a* and *RFT1* transcripts were detected in rice leaves. To balance data fluctuations, a LOG2 transformation was applied. The two points represent the results from two sequencing datasets. **D** Expression analysis of DEGs only at SD0 with elevated expression in the SAM of the double mutant (MU). **E** Expression analysis of DEGs at both SD0 and SD4, and at SD0, SD4, and SD6 with elevated expression in the SAM of the double mutant (MU). The lines connect the mean TPM values of two replicates. Black represents the wild type, and red represents the mutant

**Fig. 6:**
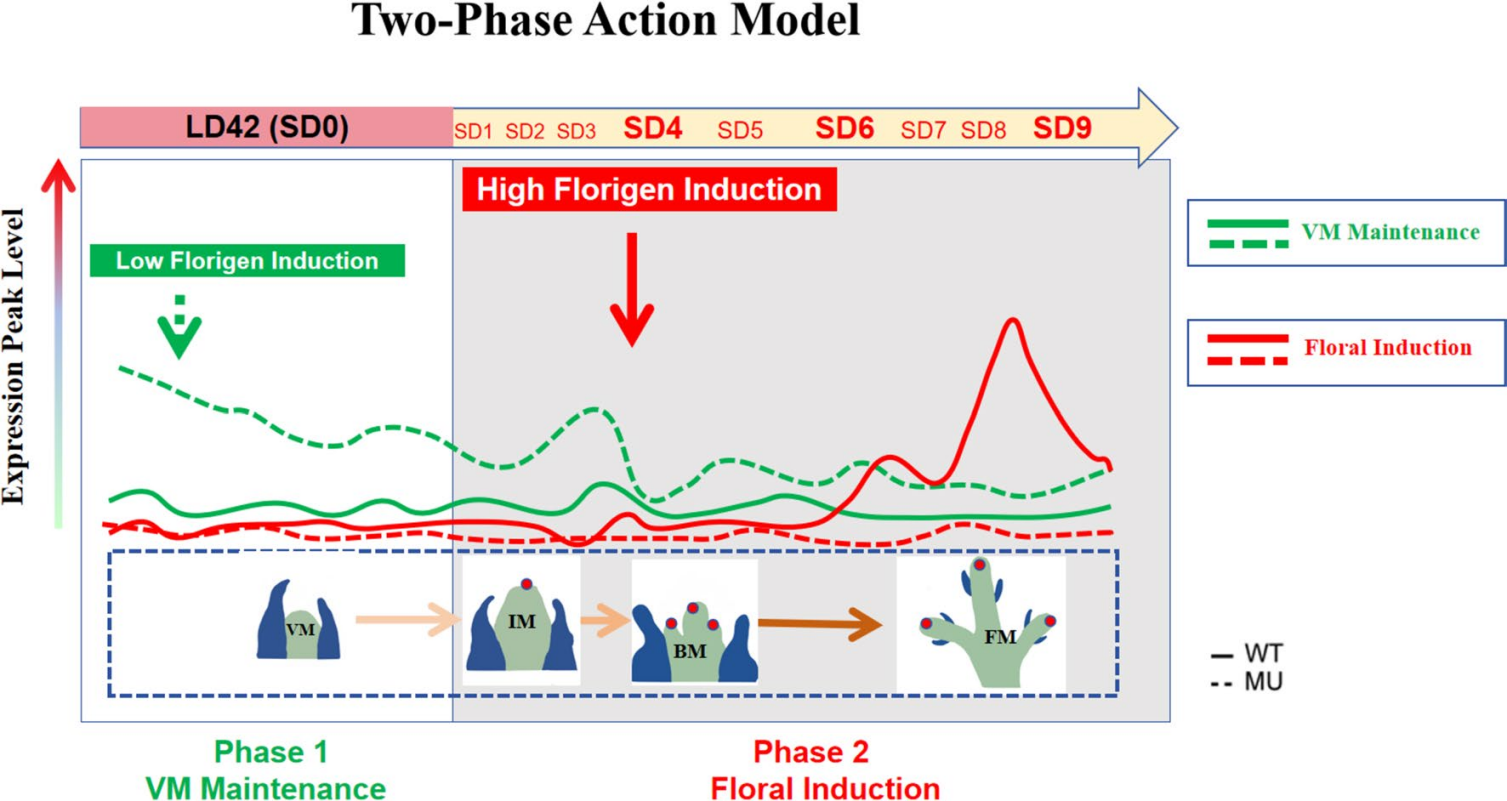
The two-phase action model of rice florigens**.** Two distinct roles of florigen genes: One is the floral induction and the other is the developmental regulation of the SAM maintenance under non-inductive conditions. Average gene expression pattern of C4 and C10 cluster genes in Fig. 2 is used as representative gene expression for the two actions in this model

The discovery of over a thousand differentially expressed genes (DEGs) at SD0 (prior to the inflorescence meristem transition) highlights a robust transcriptional activation in the double mutant line even before floral transition. This expression profile at SD0 suggests that florigen may suppress the transcription of specific genes in the wild-type plants under LD conditions. Interestingly, many of these DEGs identified at SD0 returned to wild-type expression levels following SD treatments, as evidenced by the number of DEGs specific to SD0 (1498), SD0&SD4 (691), and SD0&SD4&SD6 (153) (Fig. 5A, Fig. S1). These temporal DEG patterns reveal a clear two-phase action of florigen genes in rice: one phase operating exclusively under LD conditions and the other directly driving floral transition during SD induction (Fig. 6). The LD-specific actions of florigen genes seem to be masked under standard experimental conditions but may be influenced by environmental factors, such as distinct photoperiods (or day-lengths), ambient temperatures, and abiotic stresses.

The SD0 stage exhibited the highest number of DEGs (1498) among all SD treatment stages in the double mutant lines, emphasizing the potential role of these genes in maintaining the vegetative state of the SAM under LD conditions. Under LD conditions (e.g., SD0 or LD42), *Hd3a* and *RFT1* transcripts were detected in rice leaves (Fig. 5C), suggesting the involvement of novel complexes in response to the low levels of florigen expressions, which should be distinct from FAC (florigen activation complex) and FRC (florigen repressor complex).

From the identified 1498 and 691 DEGs, we selected transcription factors and compiled an annotated list (Fig. 5, Table S3). Based on previous knowledge, many of these transcription factors are known to regulate SAM and spikelet development, ultimately shaping inflorescence architecture. Notably, transcription factors with elevated expression levels in the double mutant lines in the list are implicated in maintaining the vegetative identity of the SAM (Fig. 5B, C, D, Table S2). Here, regulators that were differentially expressed specifically in SD0 were selected, and the expression patterns of representative differentially expressed genes in SD0&SD4 and SD0&SD4&SD6 were showcased. These representative genes, including *Oskn2*, *OsWRKY11*, and *OsbHLH068*, have all been reported to be associated with the development of the shoot apical meristem (SAM) and the final morphology of the panicle (Fig. 5D, Table S2). Especially among these regulators, anti-florigen factors, such as *OsFTL12* (Zheng et al. 2023), were also observed with increased expression levels (Fig. 5E, Table S2), suggesting their antagonistic roles in counteracting florigen activity.

The expression of these regulators during vegetative growth, which is normally suppressed (or activated) by rice florigens, may play a critical role in controlling the status of vegetative meristems under varying environmental conditions. This regulation likely sustains vegetative growth and fine-tunes the timing of floral transitions during later developmental stages. Such findings provide new insights into the hidden transcriptional mechanisms orchestrated by florigens, underscoring their importance in maintaining the vegetative state and regulating the timing of flowering in response to environmental cues.

## Discussion

### Two distinct roles of florigen genes in rice: floral induction and developmental regulation of the SAM

In this study, we comprehensively analyzed all DEGs associated with SD treatments for floral induction by comparing wild-type plants with florigen double mutant lines. Our findings suggest that the formation of inflorescence, branch, and floral meristems is governed by a more complex regulatory network than previously understood. Based on our temporal transcriptomic dataset, we propose a two-phase action model of florigens to explain these observations (Fig. 6).

### Phase 1: regulation of downstream genes under long-day conditions

Interestingly, more than a thousand genes were upregulated in the florigen double mutant lines even under long-day conditions such as SD0 (or LD42), returning to expression levels comparable to wild-type plants during floral meristem formation. These findings suggest that downstream genes of florigens are tightly regulated, remaining latent under normal SD treatments in wild-type plants. Environmental factors, such as photoperiod, ambient temperature, or abiotic stresses like drought or nitrogen deficiency, may modulate the expression of these genes.

This florigen-mediated regulation at the SAM under long-day conditions may function as a kind of "insurance" mechanism, buffering plants against unexamined environmental variables. Among these downstream genes, transcription factors such as *PINE1* and *OSH15* were highly expressed in the double mutant lines, possibly indicating a feedback mechanism that stabilizes the vegetative state in the absence of florigen (or very low levels of florigens). This regulation may involve hormonal pathways (Gomez-Ariza et al. 2019; Niu et al. 2022) and serve to prevent premature or disorganized floral meristem formation, thereby safeguarding plant fitness.

The presence of anti-florigen factors like *OsFTL12* among the upregulated transcription factors further supports an antagonistic regulatory network opposing floral transition signals in florigen-deficient backgrounds (Zheng et al. 2023). This compensatory mechanism may reflect an adaptive resilience in rice, emphasizing the significance of florigen regulation under diverse environmental conditions. For example, although *Hd3a* influences tiller number (Tsuji et al. 2015), this phenotype was not observed in the double mutant plants under the typical cultivation conditions in this study, suggesting specific environmental factors might be required to showcase such effects.

### Phase 2: initial activation of key floral regulatory genes

As anticipated, several key floral regulatory genes, including *OsMADS14/15*, *PAP2*, and *FTL-1*, were significantly upregulated in wild-type plants within four days of the SD treatment, whereas no such induction was observed in double mutant lines. At this stage, branch meristem formation was predominant (Fig. 1C, 3E). By nine days under the SD treatment, additional *MADS-box* genes, such as *SEP*-like and *AGL* genes, were upregulated, marking the onset of floral meristem initiation. These results suggest that our current RNA-seq data may lack samples capturing the transition to the inflorescence meristem (IM) stage, indicating the involvement of transcriptional regulators in addition to *OsMADS14/15* and *PAP2*.

While the exact temporal dynamics of florigen transport remain unclear, our data suggest that even low quantities of florigen molecules can exert significant regulatory effects in the SAM. This is evidenced by the detection of *Hd3a* and *RFT1* transcripts in leaves under long-day conditions, implying early production and transport of florigen proteins.

Although our RNA-seq analysis did not detect significant expressions of *Hd1* or *Ehd1* in the SAM regions, these genes regulate both flowering time and panicle development through complex networks. As demonstrated by previous results, *Hd1* and *Ehd1* function in leaf tissues by controlling florigen genes *Hd3a* and *RFT1*, which subsequently modulate the expression of panicle development genes including *OsMADS* and *RCN* factors in the SAM (Endo-Higashi and Izawa 2011). The varying expression levels of *Hd3a* and *RFT1* in leaves at the same developmental stage suggest a sophisticated mechanism where leaf-expressed regulators orchestrate both flowering time and panicle architecture through florigen-dependent pathways.

Previous studies have shown that double or multiple mutants of *FUL*-like MADS-box genes and *PAP2* fail to flower despite normal IM initiation (Kobayashi et al. 2012; Zheng et al. 2023). This supports the notion that *FUL-like* and *PAP2* genes may not be primary drivers of the vegetative-to-inflorescence meristem transition (Vicentini et al. 2023). While *OsMADS15* has been widely regarded as a key target of the florigen activation complex (FAC) in rice floral induction, its exact role warrants reevaluation. Furthermore, phenotypic analysis of triple mutants in *OsFD1*, *OsFD2*, and *OsFD4* genes revealed a delay in flowering by approximately two weeks compared to wild-type plants (Cerise, et al. 2021), whereas the florigen double mutant plants exhibited complete non-flowering phenotypes.

The involvement of other stress-responsive bZIP proteins as flowering-time regulators also raises questions about the precise nature of FAC and its formation during *Hd3a* and *RFT1* transcriptional regulation. Thus, future studies should target earlier SD-inducing samples, such as SD1, SD2, and SD3, which missed in this study, to address these gaps.

### The *miR156/529-SPL* pathway and florigen independence

Our analysis of the *miR156/529-SPL* flowering pathway revealed no differential expression of the core *SPL* genes between the wild-type and the double mutant plants. This indicates that *SPL*-mediated flowering control may function independently of florigen-mediated floral induction, consistent with prior studies (Wang et al. 2021).

In conclusion, our findings underscore the dual roles of florigen genes in floral induction and SAM maintenance regulation. The intricate interplay between florigen-mediated pathways, environmental factors, and compensatory mechanisms highlights the complexity of flowering regulation in rice and opens new avenues for future research.

### Circadian clock network independence from florigen signaling

In recent decades, plant circadian clock genes have been extensively studied for their central role in orchestrating diurnally rhythmic transcriptomes that influence a wide range of physiological processes (Hsu and Harmer 2014). However, the behavior of circadian clock genes within the SAM remains poorly understood. This study fortuitously sheds light on this underexplored aspect of plant regulatory networks.

Our findings show that the expression of major circadian clock genes, such as *OsLHY*, *OsGI*, and *OsTOC1*, is unaffected by the florigen deficiency, indicating that circadian clock regulation in the SAM operates independently of floral transition signals (Fig. 4C, D). Similarly, a large part of diurnally rhythmic genes in the SAM regions exhibited no significant changes in expression in the florigen double mutants although several genes have peak change effects by florigen genes (Fig. 4A). Gene Ontology (GO) analysis highlighted the presence of certain photosynthetic genes among these rhythmic genes; however, the expression levels of such genes in the SAM regions were very low. These observations suggest the need for a more nuanced approach when interpreting GO analyses of rhythmic genes in SAM. Thus, we do not use the GO analysis data in the study. The biological significance of the robust diurnal transcriptome dynamics in SAM, therefore, remains an open question. This resilience of the circadian clock might serve to optimize processes other than photosynthesis, such as nutrient uptake or other daily metabolic activities that support plant growth.

Interestingly, we could not detect the expression of key flowering-time genes such as *Hd1*, *Ehd1*, *Hd3a*, and *RFT1* in the SAM, despite the normal expression of *OsGI*. This suggests that tissue-specific transcriptional regulation of these core flowering-time genes is absent in the SAM regions.

In conclusion, our findings highlight two distinct roles of florigens: (1) driving the floral transition under conditions of high florigen expression and (2) potentially contributing to SAM maintenance under diverse environmental conditions (Fig. 6). Additionally, we demonstrate that the circadian clock in the SAM operates independently of florigen signaling, suggesting a robust regulatory system that persists regardless of floral transition (Fig. 5). These insights provide valuable information for further elucidating the mechanisms underlying flowering-time control and SAM regulation in rice.

## Supplementary Information

Below is the link to the electronic supplementary material.

Supplementary file 1 (PNG 651 KB)

Supplementary file 2 (PNG 1413 KB)

Supplementary file 3 (PNG 184 KB)

Supplementary file 4 (PNG 579 KB)

Supplementary file 5 (PNG 659 KB)

Supplementary file 6 (PNG 1028 KB)

Supplementary file 7 (PNG 246 KB)

Supplementary file 8 (JPG 910 KB)

Supplementary file 9 (PNG 326 KB)

Supplementary file 10 (XLSX 26 KB)

Supplementary file 10 (DOCX 33 KB)

## Data Availability

Data are contained within the article or supplementary material. Raw sequencing data have been deposited in the NCBI Sequence Read Archive (SRA) under accession number PRJNA1194998.
