# Supplementary figures and images for: Temporal transcriptome analysis reveals the two-phase action of florigens in rice flowering
Source: Theor Appl Genet. 2025 Apr 12;138(5):100. doi: 10.1007/s00122-025-04869-0 (PMC11993458; doi:10.1007/s00122-025-04869-0)

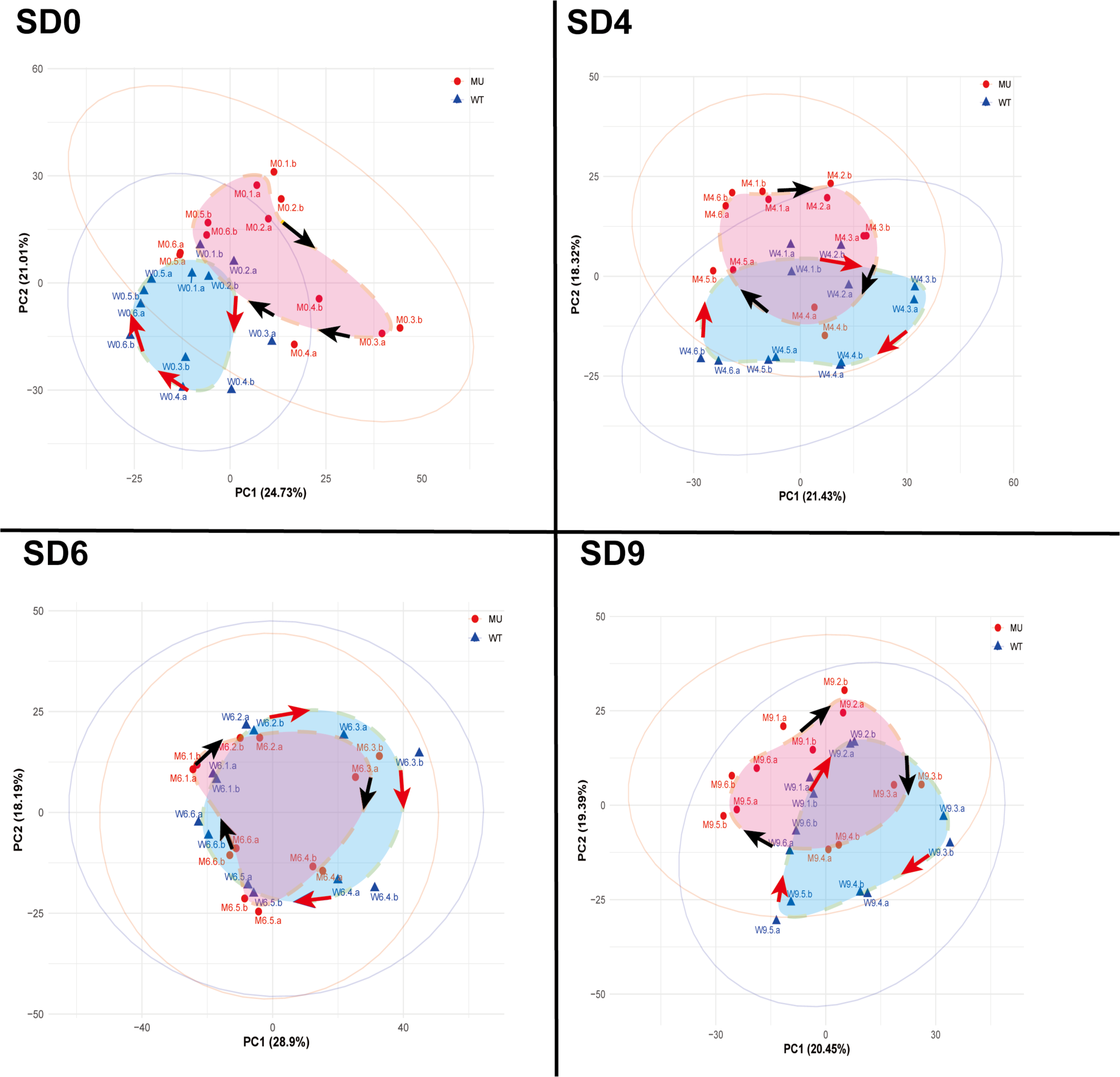

Supplement: Supplementary file 1 — (PNG 651 KB) [file 122_2025_4869_MOESM1_ESM.png]

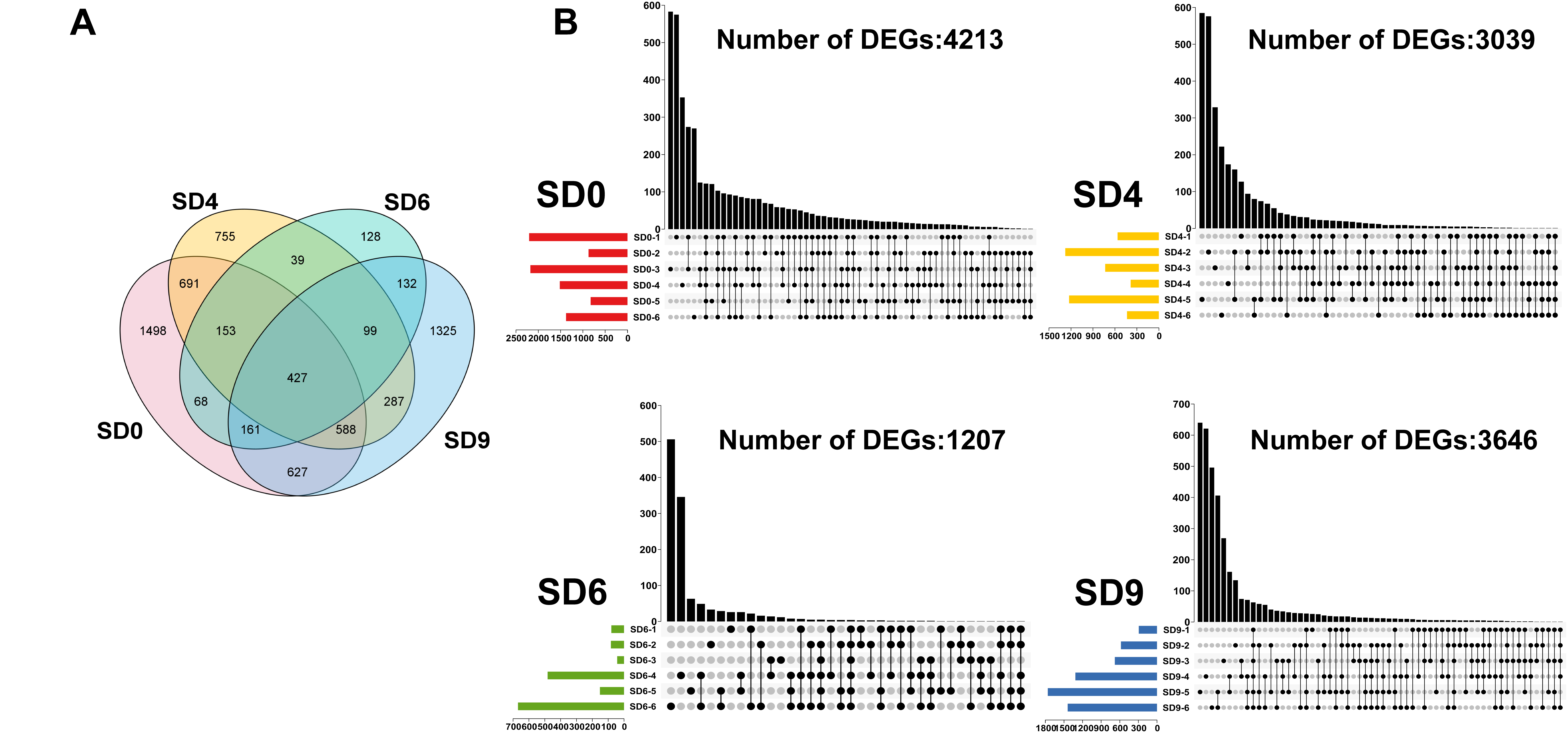

Supplement: Supplementary file 2 — (PNG 1413 KB) [file 122_2025_4869_MOESM2_ESM.png]

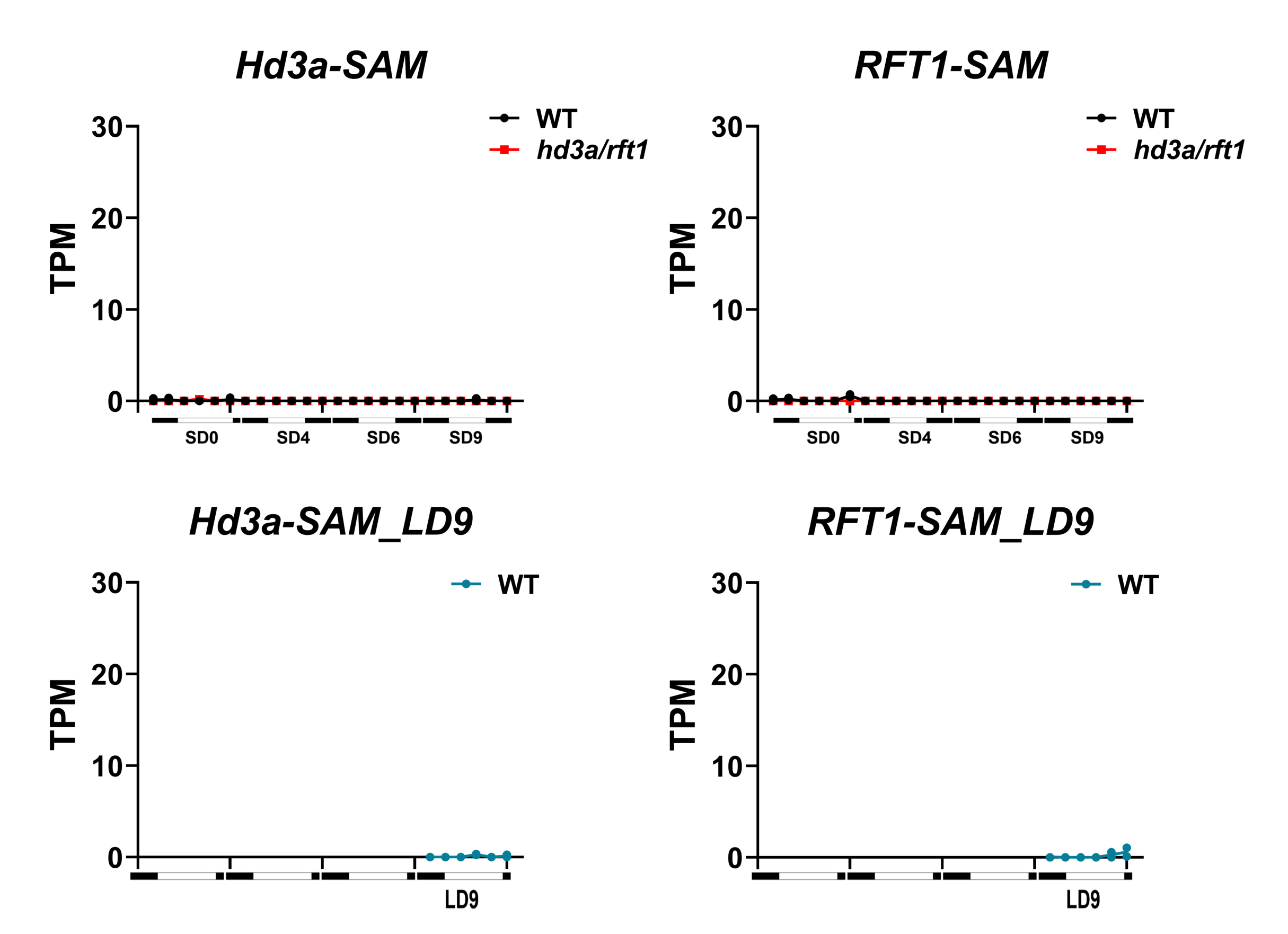

Supplement: Supplementary file 3 — (PNG 184 KB) [file 122_2025_4869_MOESM3_ESM.png]

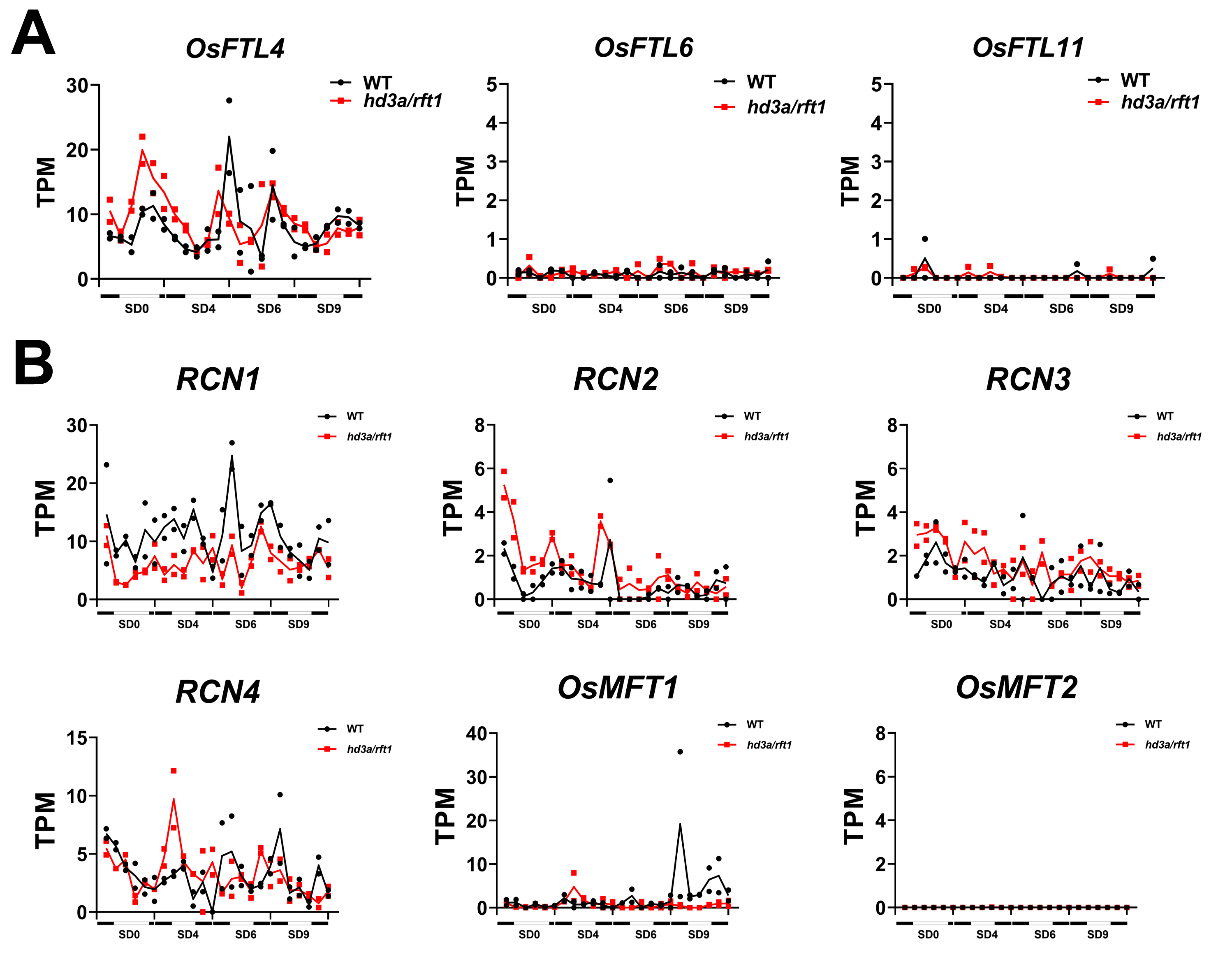

Supplement: Supplementary file 4 — (PNG 579 KB) [file 122_2025_4869_MOESM4_ESM.png]

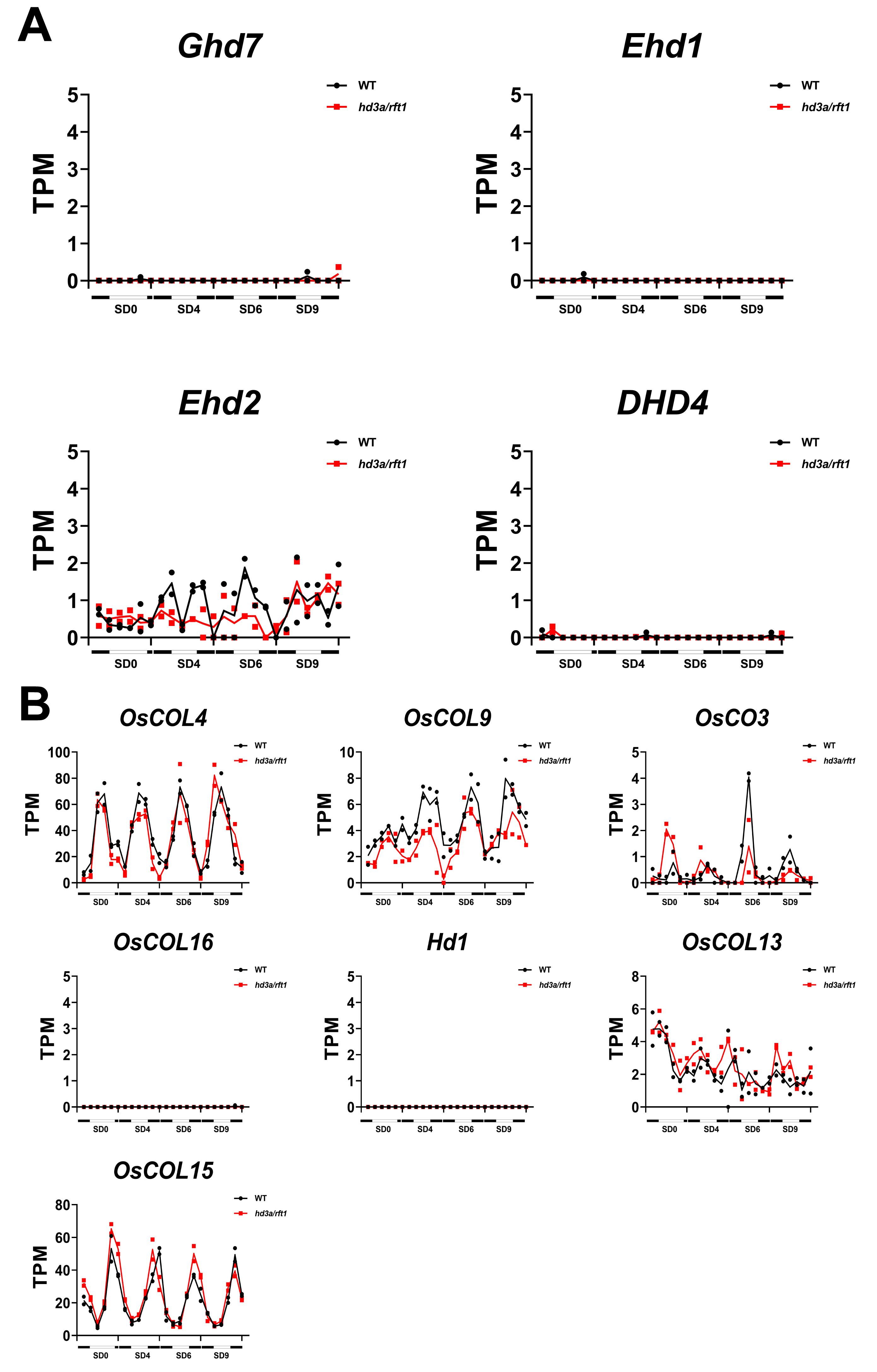

Supplement: Supplementary file 5 — (PNG 659 KB) [file 122_2025_4869_MOESM5_ESM.png]

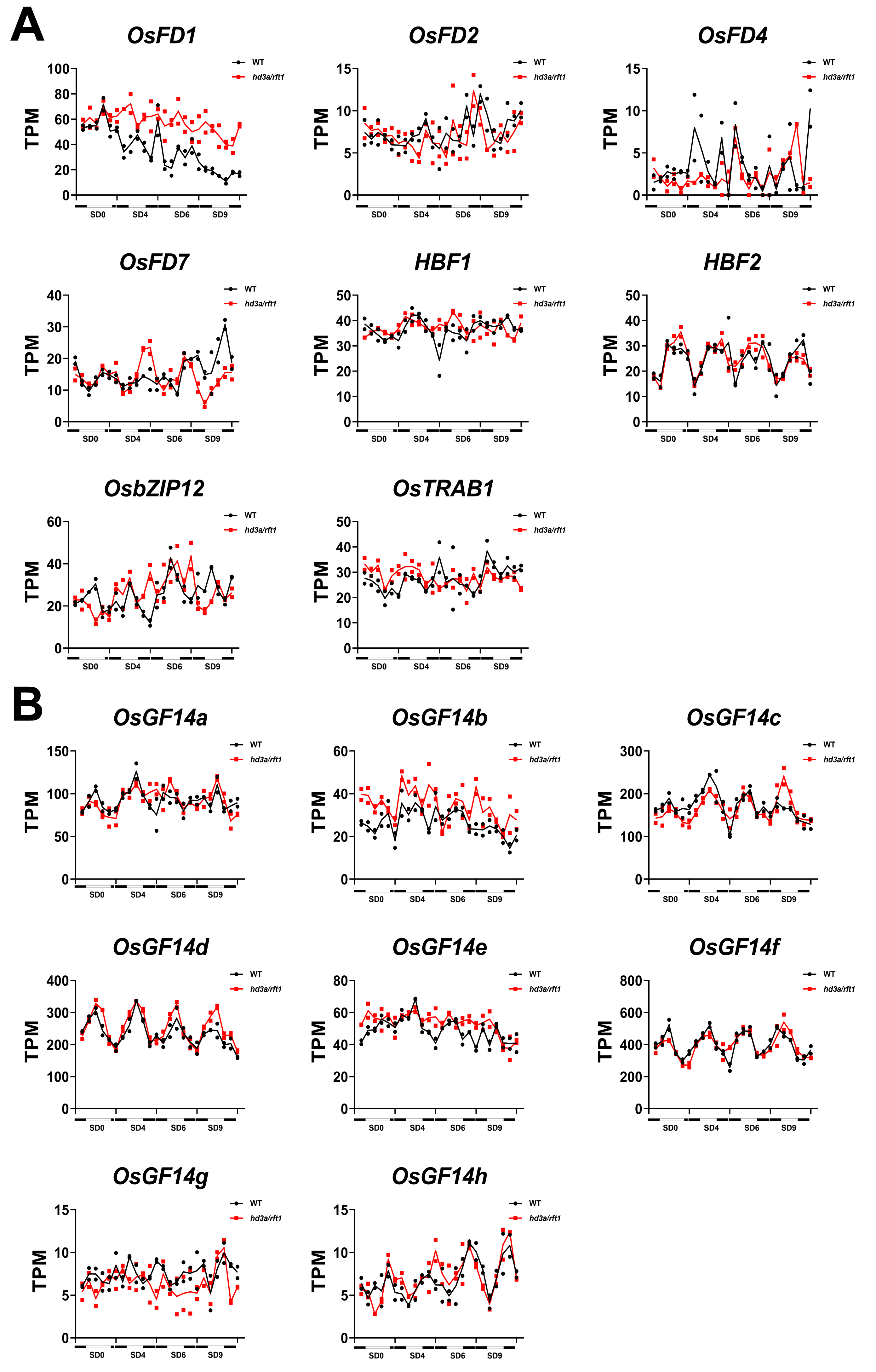

Supplement: Supplementary file 6 — (PNG 1028 KB) [file 122_2025_4869_MOESM6_ESM.png]

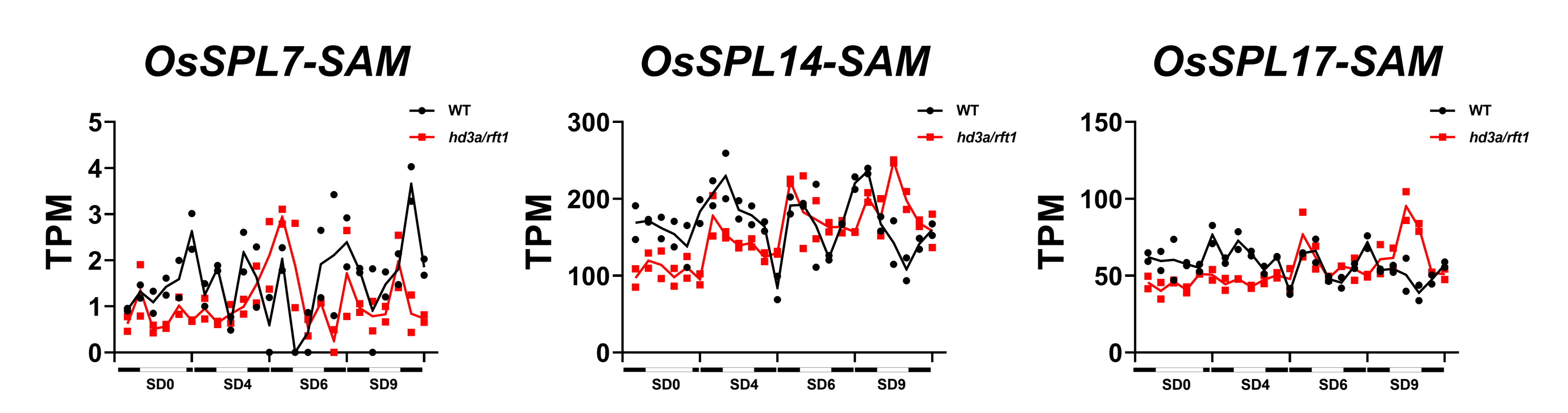

Supplement: Supplementary file 7 — (PNG 246 KB) [file 122_2025_4869_MOESM7_ESM.png]

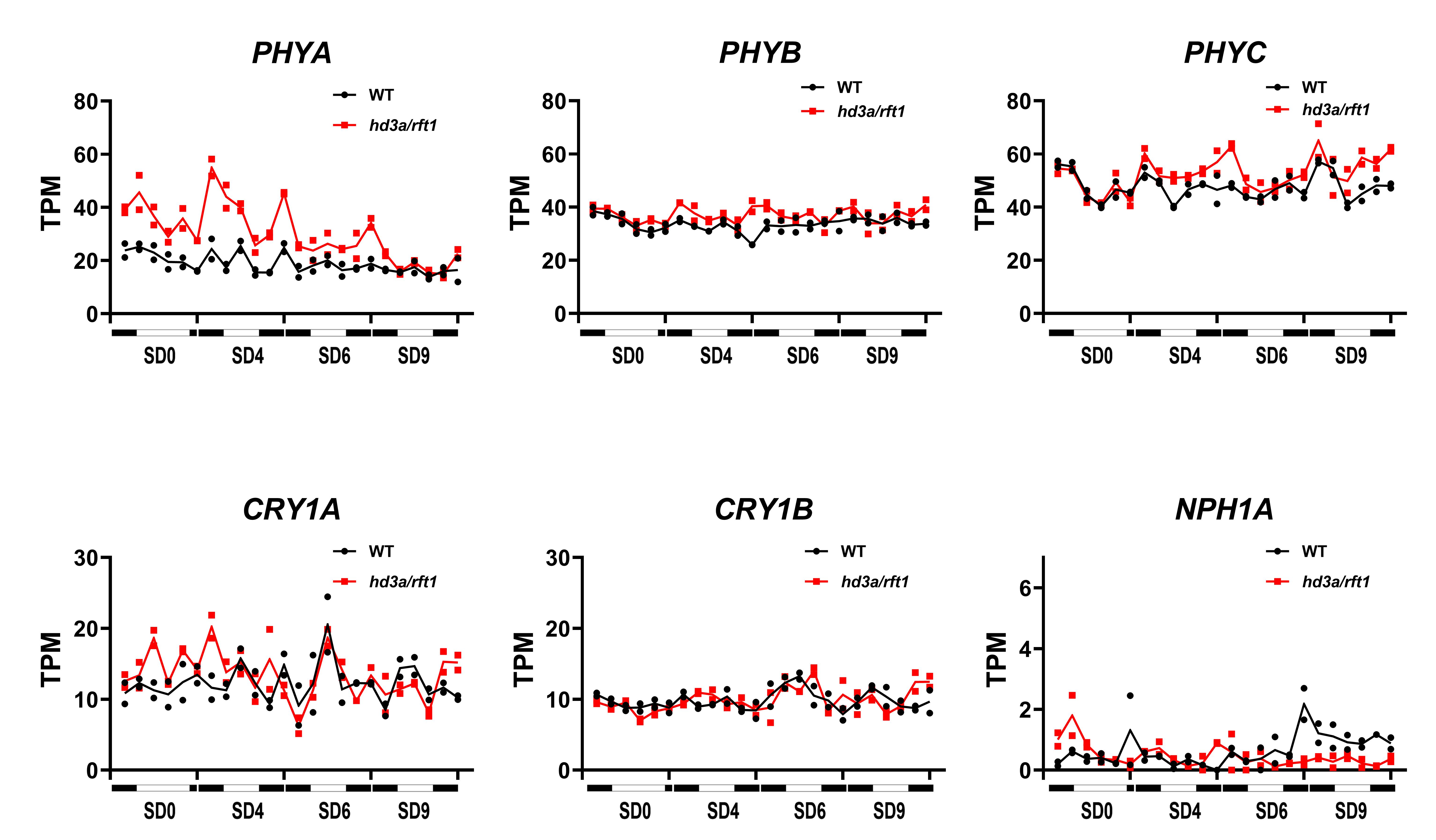

Supplement: Supplementary file 8 — (JPG 910 KB) [file 122_2025_4869_MOESM8_ESM.jpg]

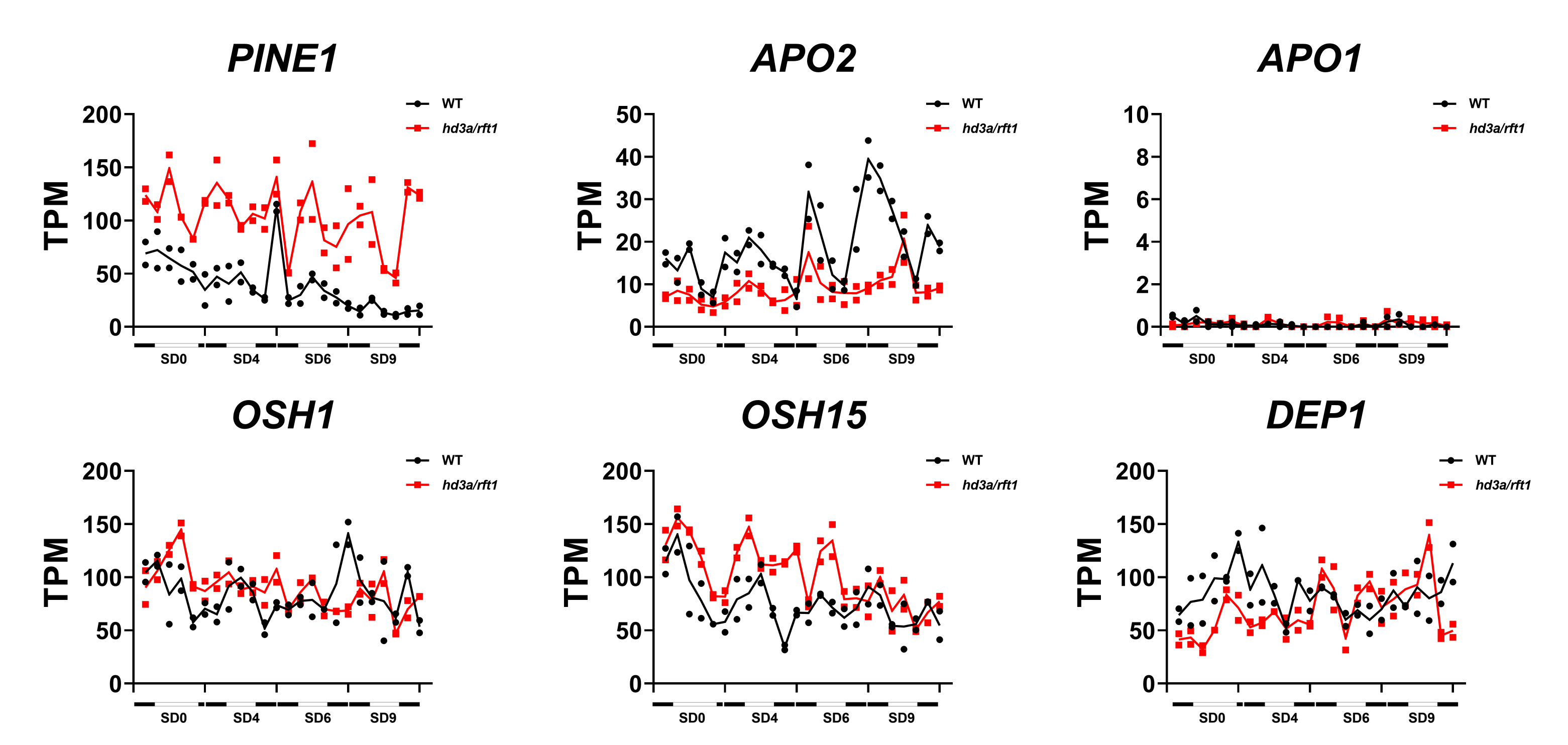

Supplement: Supplementary file 9 — (PNG 326 KB) [file 122_2025_4869_MOESM9_ESM.png]
