# Supplementary material for: Temporal transcriptome analysis reveals the two-phase action of florigens in rice flowering
Source: Theor Appl Genet. 2025 Apr 12;138(5):100. doi: 10.1007/s00122-025-04869-0 (PMC11993458; doi:10.1007/s00122-025-04869-0)
Supplement: Supplementary file 11 — Supplementary file 10 (DOCX 33 KB) [file 122_2025_4869_MOESM11_ESM.docx]

**FigS1. List of the number of DEGs identified at each time point.**

(A) The Venn diagram illustrates the quantitative relationships of DEGs across the four sampling days.

(B) The upset plot shows the distribution of differentially expressed genes at each time point over the four sampling days. SD0-1/2/3/4/5/6 corresponds to ZT-7, ZT-3, ZT1, ZT5, ZT9, and ZT13 under SD0, respectively. The same notation applies to the other days.

**FigS2. Expression analysis of florigen genes in SAM**

**FigS3. Expression analysis of *FTL(A)* and *TFL and MFT-like (B)* genes in SAM**

**FigS4. Expression analysis of *Ghd7, Ehd1/2, DHD4*(A) and *COL-like* (B) genes in SAM**

**FigS5. Expression analysis of FAC components genes in SAM. (A) bZIPs (B) GF14s**

**FigS6. Expression analysis of *SPL* genes in SAM**

**FigS7. Expression analysis of SAM maintaining genes**

**FigS8. Expression analysis of photoreceptor genes**

**FigS9. PCA analysis using rhythm＆DEG(n=1400)**

PCA analysis was performed separately for the four sampling days, revealing the separation and rhythmic variations among the samples. Circles represent the 95% confidence interval. The connections between circles are based on the mean values of two samples, and the arrows indicate the rhythmic trend over the sampling time points..

TableS1. Sequencing Quality Statistics Table

TableS2. Statistical Analysis and Annotation of Representative Genes with Increased Expression in the Double Mutant

TableS3. Information of Genes and Annotations, as well as Primers on All Genes used in This Study

Table S1

| **Sample ID** | **Sequencing Depth (Reads Count)** | **Read Length (bp)** | **Platform** | **Q30 (%)** | **Uniquely mapped reads(%)** |
| --- | --- | --- | --- | --- | --- |
| W0-1-a | 16891884 | 150 | HiSeqX_Ten | 89.59 | 91.35 |
| W0-1-b | 20357394 | 150 | HiSeqX_Ten | 89.46 | 93.40 |
| W0-2-a | 13820162 | 150 | HiSeqX_Ten | 89.71 | 91.38 |
| W0-2-b | 40626306 | 150 | HiSeqX_Ten | 89.92 | 94.10 |
| W0-3-a | 12255794 | 150 | HiSeqX_Ten | 89.80 | 93.34 |
| W0-3-b | 7955800 | 150 | HiSeqX_Ten | 90.44 | 93.04 |
| W0-4-a | 8508990 | 150 | HiSeqX_Ten | 90.11 | 92.82 |
| W0-4-b | 25068808 | 150 | HiSeqX_Ten | 90.39 | 92.28 |
| W0-5-a | 39498374 | 150 | HiSeqX_Ten | 90.23 | 92.81 |
| W0-5-b | 43551696 | 150 | HiSeqX_Ten | 90.04 | 93.03 |
| W0-6-a | 12838034 | 150 | HiSeqX_Ten | 90.43 | 93.45 |
| W0-6-b | 9472406 | 150 | HiSeqX_Ten | 89.97 | 92.17 |
| W4-1-a | 26609896 | 150 | HiSeqX_Ten | 85.57 | 93.46 |
| W4-1-b | 17437762 | 150 | HiSeqX_Ten | 85.74 | 93.61 |
| W4-2-a | 27852594 | 150 | HiSeqX_Ten | 85.40 | 93.21 |
| W4-2-b | 23712550 | 150 | HiSeqX_Ten | 85.11 | 93.25 |
| W4-3-a | 22445506 | 150 | HiSeqX_Ten | 84.97 | 91.37 |
| W4-3-b | 24462160 | 150 | HiSeqX_Ten | 85.24 | 92.88 |
| W4-4-a | 27521236 | 150 | HiSeqX_Ten | 85.28 | 92.81 |
| W4-4-b | 18063188 | 150 | HiSeqX_Ten | 85.75 | 93.12 |
| W4-5-a | 27626708 | 150 | HiSeqX_Ten | 85.38 | 91.37 |
| W4-5-b | 30150710 | 150 | HiSeqX_Ten | 85.36 | 89.13 |
| W4-6-a | 3349968 | 150 | HiSeqX_Ten | 84.75 | 93.24 |
| W4-6-b | 1098288 | 150 | HiSeqX_Ten | 84.52 | 95.59 |
| W6-1-a | 4552498 | 150 | HiSeqX_Ten | 89.05 | 92.85 |
| W6-1-b | 4466376 | 150 | HiSeqX_Ten | 89.14 | 91.87 |
| W6-2-a | 3588764 | 150 | HiSeqX_Ten | 88.11 | 93.66 |
| W6-2-b | 4109120 | 150 | HiSeqX_Ten | 87.99 | 93.56 |
| W6-3-a | 3286024 | 150 | HiSeqX_Ten | 89.05 | 87.88 |
| W6-3-b | 9241974 | 150 | HiSeqX_Ten | 89.03 | 92.71 |
| W6-4-a | 5068776 | 150 | HiSeqX_Ten | 89.42 | 91.91 |
| W6-4-b | 14174048 | 150 | HiSeqX_Ten | 89.59 | 90.90 |
| W6-5-a | 13195314 | 150 | HiSeqX_Ten | 89.42 | 92.62 |
| W6-5-b | 16982652 | 150 | HiSeqX_Ten | 89.57 | 93.86 |
| W6-6-a | 5197774 | 150 | HiSeqX_Ten | 88.17 | 93.67 |
| W6-6-b | 6686902 | 150 | HiSeqX_Ten | 87.95 | 93.89 |
| W9-1-a | 18851642 | 150 | HiSeqX_Ten | 90.87 | 94.25 |
| W9-1-b | 17530214 | 150 | HiSeqX_Ten | 90.95 | 94.83 |
| W9-2-a | 25414412 | 150 | HiSeqX_Ten | 90.79 | 90.57 |
| W9-2-b | 5391314 | 150 | HiSeqX_Ten | 90.31 | 90.64 |
| W9-3-a | 16673792 | 150 | HiSeqX_Ten | 90.28 | 91.30 |
| W9-3-b | 25692226 | 150 | HiSeqX_Ten | 90.71 | 92.86 |
| W9-4-a | 13640506 | 150 | HiSeqX_Ten | 90.47 | 90.50 |
| W9-4-b | 15653920 | 150 | HiSeqX_Ten | 90.54 | 92.90 |
| W9-5-a | 27379542 | 150 | HiSeqX_Ten | 90.43 | 93.17 |
| W9-5-b | 28986234 | 150 | HiSeqX_Ten | 90.44 | 93.63 |
| W9-6-a | 11579790 | 150 | HiSeqX_Ten | 90.66 | 94.85 |
| W9-6-b | 6126120 | 150 | HiSeqX_Ten | 90.15 | 94.46 |
| M0-1-a | 23392920 | 150 | HiSeqX_Ten | 85.04 | 93.36 |
| M0-1-b | 28834972 | 150 | HiSeqX_Ten | 85.60 | 92.24 |
| M0-2-a | 28061414 | 150 | HiSeqX_Ten | 85.83 | 93.19 |
| M0-2-b | 29881884 | 150 | HiSeqX_Ten | 86.25 | 94.50 |
| M0-3-a | 26815828 | 150 | HiSeqX_Ten | 85.71 | 91.53 |
| M0-3-b | 23774520 | 150 | HiSeqX_Ten | 85.63 | 92.30 |
| M0-4-a | 26691854 | 150 | HiSeqX_Ten | 85.65 | 93.14 |
| M0-4-b | 18509420 | 150 | HiSeqX_Ten | 85.34 | 93.28 |
| M0-5-a | 19126142 | 150 | HiSeqX_Ten | 85.19 | 93.25 |
| M0-5-b | 18459660 | 150 | HiSeqX_Ten | 85.28 | 92.10 |
| M0-6-a | 28183668 | 150 | HiSeqX_Ten | 85.30 | 90.47 |
| M0-6-b | 22413372 | 150 | HiSeqX_Ten | 85.78 | 93.82 |
| M4-1-a | 14029516 | 150 | HiSeqX_Ten | 84.62 | 74.59 |
| M4-1-b | 22104466 | 150 | HiSeqX_Ten | 85.03 | 93.30 |
| M4-2-a | 22851802 | 150 | HiSeqX_Ten | 85.34 | 88.64 |
| M4-2-b | 17470498 | 150 | HiSeqX_Ten | 85.52 | 85.56 |
| M4-3-a | 21206686 | 150 | HiSeqX_Ten | 85.54 | 93.64 |
| M4-3-b | 21101724 | 150 | HiSeqX_Ten | 85.20 | 93.25 |
| M4-4-a | 230617722 | 150 | HiSeqX_Ten | 88.16 | 94.89 |
| M4-4-b | 190738074 | 150 | HiSeqX_Ten | 87.81 | 94.26 |
| M4-5-a | 2864078 | 150 | HiSeqX_Ten | 88.91 | 93.09 |
| M4-5-b | 2900682 | 150 | HiSeqX_Ten | 88.64 | 80.01 |
| M4-6-a | 6243656 | 150 | HiSeqX_Ten | 89.07 | 57.18 |
| M4-6-b | 4392158 | 150 | HiSeqX_Ten | 89.23 | 79.31 |
| M6-1-a | 1819874 | 150 | HiSeqX_Ten | 88.98 | 92.88 |
| M6-1-b | 3348088 | 150 | HiSeqX_Ten | 88.77 | 92.79 |
| M6-2-a | 5538176 | 150 | HiSeqX_Ten | 89.28 | 92.12 |
| M6-2-b | 6478258 | 150 | HiSeqX_Ten | 89.43 | 93.72 |
| M6-3-a | 7541462 | 150 | HiSeqX_Ten | 89.20 | 92.85 |
| M6-3-b | 7647972 | 150 | HiSeqX_Ten | 89.39 | 92.59 |
| M6-4-a | 12898410 | 150 | HiSeqX_Ten | 88.67 | 92.66 |
| M6-4-b | 7386816 | 150 | HiSeqX_Ten | 88.17 | 93.56 |
| M6-5-a | 4688040 | 150 | HiSeqX_Ten | 88.99 | 93.16 |
| M6-5-b | 10577670 | 150 | HiSeqX_Ten | 89.12 | 93.82 |
| M6-6-a | 7194674 | 150 | HiSeqX_Ten | 89.50 | 86.64 |
| M6-6-b | 11409118 | 150 | HiSeqX_Ten | 89.59 | 94.44 |
| M9-1-a | 31777440 | 150 | HiSeqX_Ten | 89.62 | 63.44 |
| M9-1-b | 15359176 | 150 | HiSeqX_Ten | 90.31 | 90.78 |
| M9-2-a | 31710018 | 150 | HiSeqX_Ten | 90.16 | 87.33 |
| M9-2-b | 30915252 | 150 | HiSeqX_Ten | 90.27 | 90.20 |
| M9-3-a | 30990756 | 150 | HiSeqX_Ten | 90.15 | 93.28 |
| M9-3-b | 23397954 | 150 | HiSeqX_Ten | 90.19 | 94.02 |
| M9-4-a | 18193432 | 150 | HiSeqX_Ten | 90.41 | 93.60 |
| M9-4-b | 27971062 | 150 | HiSeqX_Ten | 90.43 | 94.17 |
| M9-5-a | 20711076 | 150 | HiSeqX_Ten | 89.95 | 84.63 |
| M9-5-b | 24023592 | 150 | HiSeqX_Ten | 90.47 | 92.85 |
| M9-6-a | 36073848 | 150 | HiSeqX_Ten | 90.48 | 84.82 |
| M9-6-b | 37256992 | 150 | HiSeqX_Ten | 90.73 | 89.58 |
| LDW9-1-a | 42387906 | 150 | HiSeqX_Ten | 90.44 | 93.33 |
| LDW9-1-b | 45155944 | 150 | HiSeqX_Ten | 90.70 | 90.55 |
| LDW9-2-a | 7108988 | 150 | HiSeqX_Ten | 90.15 | 93.87 |
| LDW9-2-b | 17544800 | 150 | HiSeqX_Ten | 90.51 | 94.03 |
| LDW9-3-a | 17729994 | 150 | HiSeqX_Ten | 90.19 | 93.24 |
| LDW9-3-b | 24703556 | 150 | HiSeqX_Ten | 90.60 | 93.21 |
| LDW9-4-a | 26320078 | 150 | HiSeqX_Ten | 89.67 | 93.67 |
| LDW9-4-b | 39078558 | 150 | HiSeqX_Ten | 90.01 | 93.64 |
| LDW9-5-a | 35827984 | 150 | HiSeqX_Ten | 89.82 | 93.49 |
| LDW9-5-b | 7877576 | 150 | HiSeqX_Ten | 89.07 | 93.26 |
| LDW9-6-a | 38571744 | 150 | HiSeqX_Ten | 89.93 | 88.04 |
| LDW9-6-b | 16971210 | 150 | HiSeqX_Ten | 89.56 | 91.74 |
| WL1a | 9624262 | 150 | HiSeqX_Ten | 89.44 | 52.14 |
| WL1b | 14100142 | 150 | HiSeqX_Ten | 89.98 | 71.05 |
| WL4a | 16787708 | 150 | HiSeqX_Ten | 89.79 | 93.86 |
| WL4b | 16701106 | 150 | HiSeqX_Ten | 89.87 | 94.31 |
| WL6a | 17979124 | 150 | HiSeqX_Ten | 90.18 | 94.00 |
| WL6b | 93467194 | 150 | HiSeqX_Ten | 89.77 | 35.96 |
| WL9a | 8459810 | 150 | HiSeqX_Ten | 89.42 | 90.87 |
| WL9b | 7420676 | 150 | HiSeqX_Ten | 89.51 | 92.22 |
| ML1a | 9351954 | 150 | HiSeqX_Ten | 89.77 | 91.09 |
| ML1b | 44395370 | 150 | HiSeqX_Ten | 89.94 | 60.28 |
| ML9a | 11746360 | 150 | HiSeqX_Ten | 89.62 | 94.81 |
| ML9b | 14042310 | 150 | HiSeqX_Ten | 89.71 | 94.75 |

Table S2

| **RAP-DB Locus** | **Gene Lable in this study** |
| --- | --- |
| **Os10g0101200** | **SCP45** |
| **Os12g0424700** | **OsCDKF2** |
| **Os08g0174500** | **Ghd8** |
| **Os01g0626400** | **OsWRKY11** |
| **Os01g0952800** | **OsbHLH056** |
| **Os01g0878700** | **OsAAP6** |
| **Os10g0515400** | **GW10** |
| **Os03g0179400** | **OsRLCK103** |
| **Os07g0182000** | **OsbZIP58** |
| **Os03g0267800** | **HDR3** |
| **Os03g0685300** | **FLO19** |
| **Os02g0196600** | **OsHMA4** |
| **Os07g0644100** | **OsbZIP60** |
| **Os04g0631600** | **OsbHLH068** |
| **Os06g0514800** | **OsThi9** |
| **Os03g0609500** | **OsLBD38** |
| **Os02g0731200** | **OsMADS57** |
| **Os05g0136200** | **OsCIPK17** |
| **Os03g0183000** | **OsAP2-125** |
| **Os02g0705500** | **OsBLR1** |
| **Os03g0181100** | **OsJAZ10** |
| **Os12g0181600** | **OsAAP15** |
| **Os02g0134200** | **OsSGL** |
| **Os03g0445700** | **OsLBD37** |
| **Os08g0157600** | **OsLHY** |
| **Os09g0532400** | **OsPRR95** |
| **Os03g0284100** | **OsPRR73** |
| **Os11g0157600** | **OsPRR59** |
| **Os07g0695100** | **OsPRR37** |
| **Os02g0618200** | **OsTOC1** |
| **Os11g0547000** | **OsFKF1** |
| **Os01g0971800** | **OsLUX** |
| **Os01g0182600** | **OsGI** |
| **Os01g0566100** | [**OsELF3-2**](https://academic.oup.com/view-large/javascript:;) |
| **Os06g0142600** | **OsELF3-1** |
| **Os10g0419200** | **Ehd2** |
| **Os10g0463400** | **Ehd1** |
| **Os02g0110100** | **DHD4** |
| **Os07g0261200** | **Ghd7** |
| **Os09g0540800** | **OsFD1** |
| **Os08g0549600** | **OsFD4** |
| **Os06g0720900** | **OsFD2** |
| **Os07g0686100** | **OsFD7** |
| **Os08g0472000** | **OsTRAB1** |
| **Os05g0489700** | **HBF1** |
| **Os01g0813100** | **HBF2** |
| **Os01g0867300** | **OsbZIP12** |
| **Os01g0886200** | **OsMADS21** |
| **Os01g0201700** | **OSMADS3** |
| **Os12g0207000** | **OsMADS13** |
| **Os05g0203800** | **OsMADS58** |
| **Os03g0752800** | **OsMADS14** |
| **Os07g0108900** | **OsMADS15** |
| **Os12g0501700** | **OsMADS20** |
| **Os07g0605200** | **OsMADS18** |
| **Os03g0215400** | **OsMADS1** |
| **Os06g0162800** | **OsMADS5** |
| **Os03g0753100** | **OsMADS34** |
| **Os09g0507200** | **OsMADS8** |
| **Os08g0531700** | **OsMADS7** |
| **Os03g0122600** | **OsMADS50** |
| **Os10g0536100** | **OsMADS56** |
| **Os06g0712700** | **OsMADS16** |
| **Os01g0726400** | **OsMADS32** |
| **Os04g0580700** | **OsMADS17** |
| **Os02g0682200** | **OsMADS6** |
| **Os08g0531900** | **OsMADS37** |
| **Os01g0922800** | **OsMADS51** |
| **Os05g0423400** | **OsMADS4** |
| **Os02g0170300** | **OsMADS29** |
| **Os02g0761000** | **OsMADS22** |
| **Os06g0217300** | **OsMADS55** |
| **Os02g0104200** | **OsMADS60** |
| **Os06g0665400** | **APO1** |
| **Os04g0598300** | **APO2** |
| **Os07g0129700** | **OSH15** |
| **Os03g0727000** | **OSH1** |
| **Os12g0617000** | **PINE1** |
| **Os08g0430500** | **GF14c** |
| **Os02g0580300** | **OsGF14e** |
| **Os03g0710800** | **OsGF14f** |
| **Os04g0462500** | **OsGF14b** |
| **Os08g0480800** | **OsGF14a** |
| **Os11g0546900** | **OsGF14d** |
| **Os01g0209200** | **OsGF14g** |
| **Os11g0609600** | **OsGF14h** |
| **Os06g0157700** | **Hd3a** |
| **Os06g0157500** | **RFT1** |
| **Os01g0218500** | **OsFTL1** |
| **Os06g0552900** | **OsFTL12** |
| **Os05g0518000** | **OsFTL10** |
| **Os09g0513500** | **OsFTL4** |
| **Os01g0202700** | **OsFTL8** |
| **Os04g0488400** | **OsFTL6** |
| **Os12g0232300** | **OsFTL7** |
| **Os11g0293800** | **OsFTL11** |
| **Os05g0468800** | **OsFTL14** |
| **Os09g0441900** | **DEP1** |
| **Os01g0111600** | **OsMFT2** |
| **Os06g0498800** | **OsMFT1** |
| **Os02g0531600** | **RCN2** |
| **Os11g0152500** | **RCN1** |
| **Os04g0411400** | **RCN4** |
| **Os12g0152000** | **RCN3** |
| **Os09g0491532** | **OsSPL17** |
| **Os04g0551500** | **OsSPL7** |
| **Os08g0509600** | **OsSPL14** |
| **Os06g0275000** | **Hd1** |
| **Os08g0536300** | **OsCOL15** |
| **Os03g0711100** | **OsCOL9** |
| **Os06g0264200** | **OsCOL16** |
| **Os02g0610500** | **OsCOL4** |
| **Os07g0667300** | **OsCOL13** |
| **Os09g0240200** | **OsCO3** |
| **Os10g0403000** | **PLA1** |
| **Os05g0578900** | **NL1** |
| **Os03g0719800** | **PHYA** |
| **Os03g0309200** | **PHYB** |
| **Os03g0752100** | **PHYC** |
| **Os02g0573200** | **CRY1A** |
| **Os04g0452100** | **CRY1B** |
| **Os12g0101800** | **NPH1A** |

Table S3

| **ID** | **Lable** | **category** | **Max Log2FC** | **Function** | **Reference** | **Title** |
| --- | --- | --- | --- | --- | --- | --- |
| Os10g0101200 | **SCP45** | SD0 | 4.45 | Regulation of grain filling and seed germination | Li et al. 2016 | **Serine carboxypeptidase 46 Regulates Grain Filling and Seed Germination in Rice (Oryza sativa L.)** |
| Os12g0424700 | **OsCDKF2** | SD0 | 4.44 | Regulation of grain filling and grain size | Zhao Y et al. 2023 | **The miR167-OsARF12 module regulates rice grain filling and grain size downstream of miR159** |
| Os08g0174500 | **Ghd8** | SD0 | 3.62 | Flowering time, Short-day promotion, Long-day repression | Wei et al., 2010 | **DTH8 Suppresses Flowering in Rice, Influencing Plant Height and Yield Potential Simultaneously** |
| Os01g0626400 | **OsWRKY11** | SD0 | 3.07 | Control of flowering time and plant height | Cai et al. 2014 | **Dlf1, a WRKY Transcription Factor, Is Involved in the Control of Flowering Time and Plant Height in Rice** |
| Os01g0952800 | **OsbHLH056** | SD0 | 2.80 | Fe translocation to grain during seed maturation | Ogo et al., 2011 | **OsIRO2 is responsible for iron utilization in rice and improves growth and yield in calcareous soil** |
| Os01g0878700 | **OsAAP6** | SD0 | 2.79 | Regulation of grain protein content and nutritional quality | Peng et al. 2014 | **OsAAP6 functions as an important regulator of grain protein content and nutritional quality in rice** |
| Os02g0531600 | **RCN2** | SD0 | 2.59 | Control of flowering time | Nakagawa et al. 2002 | **Overexpression of *RCN1* and *RCN2*, rice *TERMINAL FLOWER 1/CENTRORADIALIS* homologs, confers delay of phase transition and altered panicle morphology in rice** |
| Os10g0515400 | **GW10** | SD0 | 2.47 | Control of grain size and grain number | Zhan et al. 2021 | ***GW10*, a member of P450 subfamily regulates grain size and grain number in rice** |
| Os03g0179400 | **OsRLCK103** | SD0 | 2.26 | Regulator of grain yield | Ramegowda et al. 2014 | **Rice *GROWTH UNDER DROUGHT KINASE* Is Required for Drought Tolerance and Grain Yield under Normal and Drought Stress Conditions** |
| Os07g0182000 | **OsbZIP58** | SD0 | 1.91 | Grain filling | Kang L et al. 2022 | **Genome-Wide Identification of R2R3-MYB Transcription Factor and Expression Analysis under Abiotic Stress in Rice** |
| Os03g0267800 | **HDR3** | SD0 | 1.72 | Positive regulation of grain size and weight | Gao et al. 2021 | **The ubiquitin-interacting motif-type ubiquitin receptor HDR3 interacts with and stabilizes the histone acetyltransferase GW6a to control the grain size in rice** |
| Os03g0685300 | **FLO19** | SD0 | 1.59 | Regulation of grain quality | Lou et al. 2021 | **FLOURY ENDOSPERM19 encoding a class I glutamine amidotransferase affects grain quality in rice** |
| Os02g0196600 | **OsHMA4** | SD0 | 1.39 | Control of Cu accumulation in rice grain | Huang et al. 2016 | **A heavy metal P-type ATPase OsHMA4 prevents copper accumulation in rice grain** |
| Os07g0644100 | **OsbZIP60** | SD0 | 1.38 | Regulation of grain chalkiness | Cao et al. 2022 | ***OPAQUE3*, encoding a transmembrane bZIP transcription factor, regulates endosperm storage protein and starch biosynthesis in rice** |
| Os04g0631600 | **OsbHLH068** | SD0 | 1.36 | Control of flowering | Chen et al. 2017 | **The function of *OsbHLH068* is partially redundant with its homolog, *AtbHLH112*, in the regulation of the salt stress response but has opposite functions to control flowering in *Arabidopsis*** |
| Os06g0514800 | **OsThi9** | SD0＆SD4 | 9.70 | Alleviation of Cd toxicity and accumulation in rice grain | Liu X et al. 2023 | **Plant Defensin-Dissimilar Thionin OsThi9 Alleviates Cadmium Toxicity in Rice Plants and Reduces Cadmium Accumulation in Rice Grains** |
| Os03g0609500 | **OsLBD38** | SD0＆SD4 | 5.30 | Regulation of heading date and yield | Li et al. 2017 | **OsLBD37 and OsLBD38, two class II type LBD proteins, are involved in the regulation of heading date by controlling the expression of *Ehd1* in rice** |
| Os06g0552900 | **OsFTL12** | SD0＆SD4 | 3.28 | Modulation of the heading date and plant architecture | Zheng et al. 2023 | **OsFTL12, a member of FT-like family, modulates the heading date and plant architecture by florigen repression complex in rice** |
| Os02g0731200 | **OsMADS57** | SD0＆SD4 | 2.82 | Control of tillering | Guo et al. 2013 | **The interaction between OsMADS57 and OsTB1 modulates rice tillering via DWARF14** |
| Os05g0136200 | **OsCIPK17** | SD0＆SD4 | 1.45 | Grain filling, Regulation of grain development | Gao et al. 2022 | **Functional Analysis of *OsCIPK17* in Rice Grain Filling** |
| Os03g0183000 | **OsAP2-125** | SD0＆SD4＆SD6 | 3.36 | Positive regulator of grain length | Yu et al. 2017 | ***OsLG3* contributing to rice grain length and yield was mined by Ho-LAMap** |
| Os02g0705500 | **OsBLR1** | SD0＆SD4＆SD6 | 1.60 | Regulation of leaf angle and grain length | Jang et al. 2021 | **Modulation of Rice Leaf Angle and Grain Size by Expressing *OsBCL1* and *OsBCL2* under the Control of *OsBUL1* Promoter** |
| Os03g0181100 | **OsJAZ10** | SD0＆SD4＆SD6＆SD9 | 3.86 | Grain size | HAKATA et al. 2012 | **Overexpression of TIFY genes promotes plant growth in rice through jasmonate signaling** |
| Os12g0181600 | **OsAAP15** | SD0＆SD4＆SD6＆SD9 | 2.72 | Mediation of panicle branching and grain yield | Yang X et al.2023 | **OsAAP15, an amino acid transporter in response to nitrogen concentration, mediates panicle branching and grain yield in rice** |
| Os02g0134200 | **OsSGL** | SD0＆SD4＆SD6＆SD9 | 2.14 | Regulation of stress-tolerance and grain length | Wang et al. 2016 | ***OsSGL*, a novel pleiotropic stress-related gene enhances grain length and yield in rice** |
| Os12g0617000 | **PINE1** | SD0＆SD4＆SD6＆SD9 | 1.80 | Suppression of internode elongation | Nagai et al. 2020 | **Antagonistic regulation of the gibberellic acid response during stem growth in rice** |
| Os03g0445700 | **OsLBD37** | SD0＆SD4＆SD6＆SD9 | 1.31 | Regulation of heading date and yield | Li et al. 2017 | **OsLBD37 and OsLBD38, two class II type LBD proteins, are involved in the regulation of heading date by controlling the expression of *Ehd1* in rice** |

Table S3 primers

| OsGI-F | gtgaattgTAATACGACTCACTATAGGG TTCGCTGGTTGACTCTCCAC |
| --- | --- |
| OsGI-R | GCTCACCTGACCCTTACACC |
| OsLHY-F | gtgaattgTAATACGACTCACTATAGGG GTGGCTTCCTTGAGCACTCT |
| OsLHY-R | CCAATGGTGCAGGGAAGGAT |
| MADS15-F | gtgaattgTAATACGACTCACTATAGGG ATTGAGGTGGCTCAGCATCC |
| MADS15-R | GCTGAATCCGAGAGTGAGGG |
| OsMADS34-F | GATCAAGAAAGACTCAGGTA |
| OsMADS34-R | gtgaattgTAATACGACTCACTATAGGGttggcaagcacttaattagc |
